# A critical role for iron and zinc homeostatic systems in the evolutionary adaptation of *Escherichia coli* to metal restriction

**DOI:** 10.1099/mgen.0.001153

**Published:** 2023-12-06

**Authors:** Joy R. Paterson, Joshua M. Wadsworth, Ping Hu, Gary J. Sharples

**Affiliations:** ^1^​ Department of Biosciences, Durham University, Durham, UK; ^2^​ Procter and Gamble, Mason Business Center, Cincinnati, Ohio 45040, USA

**Keywords:** acid tolerance, *cadC*, chelating agents, *fepA*, metal homeostasis, *yeiR*

## Abstract

Host nutritional immunity utilizes metal deprivation to help prevent microbial infection. To investigate bacterial adaptation to such restrictive conditions, we conducted experimental evolution with two metal sequestering agents. Ethylenediaminetetraacetic acid (EDTA) and diethylenetriamine pentamethylene phosphonic acid (DTPMP) were selected as ligands because they differentially affect cellular levels of iron, manganese and zinc in *Escherichia coli *. Mutants of *E. coli* strain BW25113 were isolated after cultivation at sub-minimum inhibitory concentration (MIC) chelant levels and genetic changes potentially responsible for tolerance were identified by whole-genome sequencing. In EDTA-selected strains, mutations in the promoter region of *yeiR* resulted in elevated gene expression. The *yeiR* product, a zinc-specific metallochaperone, was confirmed to be primarily responsible for EDTA resistance. Similarly, in two of the DTPMP-selected strains, a promoter mutation increased expression of the *fepA-entD* operon, which encodes components of the ferric-enterobactin uptake pathway. However, in this case improved DTPMP tolerance was only detectable following overexpression of FepA or EntD *in trans*. Additional mutations in the *cadC* gene product, an acid-response regulator, preserved the neutrality of the growth medium by constitutively activating expression of the *cadAB* regulon. This study uncovers specific resistance mechanisms for zinc and iron starvation that could emerge by selection against host nutritional immunity or competition with heterologous metallophores. It also provides insight into the specific metals affected by these two widely used chelators critical for their antibacterial mode of action.

## Data Summary

All supporting data and protocols have been provided within the article or through supplementary data files. Twenty supplementary figures and two supplementary tables are available with the online version of this article. Genomics sequence datasets related to this article are available at the National Center for Biotechnology Information (NCBI) Sequence Read Archive (SRA) database (BioProject PRJNA989548) and can be accessed here: https://www.ncbi.nlm.nih.gov/bioproject/989548

**Impact Statement**

Disrupting cellular metal equilibrium, either by insufficiency or surplus, can be harnessed to restrict bacterial proliferation. Chelating agents that deprive bacteria of essential metal ions present an alternative approach to combat increasingly antibiotic-resistant pathogens. As part of an evaluation of their utility in this regard, we investigated the potential of *Escherichia coli* to develop resistance to two metal-sequestering compounds that target different metals. Mutants were isolated after exposure to low levels of each chelator and affected genes were identified by whole-genome sequencing. The upregulated expression of two genes, *yeiR* and *fepA*, involved in zinc and iron utilization, was found to contribute to tolerance. In addition, mutations affecting a transcriptional regulator, *cadC*, caused sustained activation of an acid tolerance pathway; this seems to confer a selective advantage on *E. coli* growing for prolonged periods in unbuffered media by maintaining their surroundings at a neutral pH. The results establish zinc and iron as key targets of the two compounds and highlight how tolerance can arise by altering levels of metal delivery or acquisition systems. Although tolerance can occur, the combination of two chelating agents with differing metal preferences should help prevent the development of resistance.

## Introduction

Nutritional immunity is an intrinsic component of the vertebrate innate immune system and functions to deprive micro-organisms of essential metal nutrients and thus safeguard against infection [1]. Iron, manganese and zinc are selectively targeted for removal as they are vital for bacterial growth and survival. Fe(III) is bound tightly in metalloproteins such as haemoglobin and in storage proteins such as ferritin in the cytosol to ensure that it is inaccessible to pathogens [2, 3]. In the phagolysosomal compartment of macrophages, the integral membrane transporter NRAMP1 actively exports both iron and manganese to deprive bacterial pathogens of these metals [4–6]. The heterodimeric protein calprotectin is a major factor in restricting availability of manganese, zinc, iron and nickel [7, 8]. Metallothionein, glutathione, psoriasin and calgranulin C contribute significantly to zinc sequestration during infection [9–11]. Bacteria counteract host-mediated metal starvation by upregulating metal selective importers and synthesizing and exporting their own chelators to assist in metal uptake. For instance, siderophores such as enterobactin have evolved to acquire iron [1, 12]. Competition between bacteria for metal ions using different autogenous and natural chelators is increasingly recognized as important in mixed species populations, including at the host–microbiome interface [13, 14].

Synthetic chelating agents bind metals with high affinity, share analogous chemistries with biologically synthesized siderophores and can impose similar restrictive conditions that resemble those dictated by nutritional immunity. Ethylenediaminetetraacetic acid (EDTA) is a well-known metal chelating agent that has a wide range of applications, from use as a preservative in consumer products, such as soaps and shampoos, to commercially available wound dressings to manage infection [15]. It is a hexadentate ligand, binding metal ions at its two nitrogen and four of its carboxylate oxygen sites. EDTA exerts its antimicrobial effects through metal restriction [16]. It can increase Gram-negatives’ permeability, probably by removing divalent cations that stabilize the bacterial outer membrane [17–19]. As such, it serves as a potentiator of antibiotic activity [20, 21] and can affect biofilm stability [22]. Most recently, EDTA has been shown to reduce cellular manganese concentrations in *Escherichia coli* by 5- to 15-fold, coupled with lesser reductions in zinc and iron [16].

Diethylenetriamine pentamethylene phosphonic acid (DTPMP) is a synthetic nitrogenous polyphosphonate that binds tightly to di- and trivalent metal ions, including iron, copper and zinc [23]. It acts as a multidentate ligand, binding metals via three tertiary amine groups within the backbone and three or more pendant phosphonate groups. DTPMP is employed in the production of detergents and as a general-purpose scale inhibitor in wastewater treatment [24]. Few studies have investigated the effect of DTPMP on bacterial growth; however, it is known to cause a reduction in cellular iron, coupled with an increase in manganese in *E. coli* [16].

To examine bacterial adaptation to metal restriction and gain further insight into the cellular targets or subsidiary effects of chelating agents, we attempted to isolate resistant mutants of *E. coli* as a representative Gram-negative bacterial species. The results reveal that chelant-mediated zinc and iron deprivation can be tolerated by boosting levels of a zinc metallochaperone, YeiR, or ferric-enterobactin production and import via FepA and EntD, in keeping with these metals being critical for bacterial growth. Additional alterations in CadC that constitutively activate the *cadAB* acid tolerance pathway benefit *E. coli* experiencing prolonged culture in unbuffered media.

## Methods

### Isolation of chelant-tolerant mutants and whole-genome sequence analysis

*E. coli* strain BW25113 [*rrnB3* Δ*lacZ4787 hsdR514* Δ(*araBAD*)*567* Δ(*rhaBAD)568 rph-1*] was initially cultivated in Luria–Bertani (LB) broth in 1 ml volumes in the presence of low concentrations of EDTA and DTPMP and repeatedly sub-cultured daily for 15 days at 37 °C. Cultures from this population were divided and transferred to 96-well microtitre plates (a 25 µl inoculum in 225 µl of media), which allowed testing of additional (higher) chelant concentrations. These were grown for a further 14 days. Single colonies were isolated at days 15 and 29, frozen stocks created and selected strains sequenced. A control without chelant was grown in parallel over the 29 day period and a purified isolate, JN186, recovered from this population.

For whole-genome sequencing by MicrobesNG a single colony of each isolate was streaked on an LB agar plate, incubated for 24 h and the colonies harvested using a sterile loop (~5×10^9^ cells) in 100 µl of phosphate-buffered saline (PBS). Lysozyme (0.1 mg ml^−1^) and RNase A (0.1 mg ml^−1^) were added to 5–40 µl of the cell suspension and incubated for 25 min at 37 °C. Proteinase K (0.1 mg ml^−1^) and SDS (0.5 % v/v) were added and samples were incubated for 5 min at 65 °C to promote lysis. Genomic DNA was purified using an equal volume of SPRI beads (Beckman Coulter) and quantified using a Quant-iT dsDNA HS kit (Thermo Fisher Scientific). Genomic DNA libraries were prepared using the Nextera XT Library Prep kit (Illumina, San Diego, USA) essentially following the manufacturer’s instructions. The whole-genome sequence of each strain was determined, including the wild-type (wt), using an Illumina MiSeq platform with 30× coverage using a 250 bp paired end protocol. Reads were adapter-trimmed using Trimmomatic 0.30 with a sliding window quality cut-off of Q15. *De novo* assembly was performed on samples using SPAdes version 3.12 [25] and contigs annotated using Prodigal 2.6 [26] and Prokka 1.11 [27]. A variant-calling pipeline was modified from NYU genomics core using GATK [28], as described elsewhere (https://gencore.bio.nyu.edu/variant-calling-pipeline/), and variants between the parental strains and mutant derivatives were annotated by SnpEff version 4.3 [29].

### Bacterial growth inhibition by chelants

Chelating agents were obtained from commercial sources and are listed in Table S1 (available in the online version of this article). Carvacrol and triclosan were purchased from Merck. Appropriate vehicle controls (water, DMSO or ethanol) were performed in parallel for all growth experiments involving these chelants. *E. coli* K-12 BW25113 and deletion–insertion derivatives were obtained from the Keio collection [30], JW4092 (Δ*cadA::kan*), JW4094 (Δ*cadC::kan*), JW2161 (Δ*yeiR::kan*) and JW5790 (Δ*yjiA::kan*). T7 expression constructs in pET22b(+) encoding *E. coli cadC* WT and mutant derivatives (T236A, M448V, Y453H), *fepA*, *entD* and *yeiR* were codon-optimized and synthesized by GenScript. For microdilution minimum inhibitory concentration (MIC) assays, *E. coli* cultures were grown in LB media (Lennox, Sigma Aldrich) in an orbital shaker (Stuart) at 37 °C to an OD_600 nm_ of 0.07, equivalent to a 0.5 MacFarland standard (240 µM BaCl_2_ in 0.18 M H_2_SO_4_ aq) and diluted 10-fold in LB broth for use as an inoculum [31, 32]. The diluted culture (50 µl, 5×10^6^ c.f.u. ml^−1^) was then transferred into a 96-well, round-bottomed microtitre plate (Sarstedt). Chelants from stock samples were diluted to yield a twofold series in LB broth and 50 µl mixed with the diluted inoculum to give a final volume of 100 µl. Plates were incubated at 37 °C with shaking at 130 r.p.m. for 16 h and absorbance at OD_600 nm_ was monitored on a Spectrostar Nano plate reader. MICs were defined as the minimum concentration of chelant needed to inhibit growth by >90 % relative to controls. Growth inhibition assays were also performed with *E. coli* B strain BL21-AI (F^–^ *ompT gal dcm lon hsdSB(rB^–^ mB^–^) [malB^+^]K-12(λS) araB::T7RNAP-tetA*) carrying pYeiR, pFepA and pEntD constructs and cells induced for target gene expression by addition of IPTG and arabinose.

### β-galactosidase assays to monitor *cadA-lacZ* promoter activity

Promoter activity assays were performed with the *E. coli* K-12 *cadA* reporter strain EP314 (*cadC1*∷Tn*10 cadA′*∷*lacZ* [33]), a derivative of MC4100 [*araD139* Δ(*lacIZYA-argF*)*U169 rpsL150 relA1 flhD5301 deoC1 fruA25 rbsR22*] [34]. EP314 was transformed with pCadC constructs alongside a pET22b(+) vector control essentially as described previously [35]. Bacteria were cultivated in LB broth at pH 7 containing 100 µg ml^−1^ ampicillin for 16 h and inoculated into LB at pH 5 with 10 mM lysine in sterile cuvettes (1 ml) in the presence or absence of 50 mM cadaverine to an OD_600 nm_ of 0.5. Eighty microlitres of culture was transferred to a 96-well microtitre plate followed by addition of 120 µl master mix (60 mM Na_2_HPO_4_, 40 mM NaH_2_PO_4_, 10 mM KCl, 1 mM MgSO_4_, 36 mM β-mercaptoethanol, 166 µl ml^−1^ T7 lysozyme, 1.1 mg ml^−1^ ONPG and 6.7 % PopCulture Reagent obtained from Merck Millipore). This was then transferred to a SPECTROstar Nano absorbance plate reader (BMG LABTECH) set to 30 °C with shaking at 400 r.p.m., with absorbance readings taken at 420 and 550 nm every minute for 3 h. Miller units were calculated using the following equation 1000×[(OD_420 nm_−1.75×OD_550 nm_)]/(*T*×*V*×OD_600 nm_) where *T*= time (min) and *V*=volume (ml, 0.2).

### Quantitative polymerase chain reaction (qPCR) assays to monitor *yeiR*, *fepA* and *cadA* promoter activity and cellular responses to iron (*fepD*), manganese (*mntS*) and zinc (*znuA*) starvation

Experiments were performed essentially as described previously [36]. Bacteria were cultivated in LB media (5 ml) to early log phase (OD_600 nm_ 0.3) with chelant added, if required, and incubated at 37 °C for 30 min before mixing 1 ml of culture with 2 ml of RNAprotect Bacteria Reagent (Qiagen). RNA was extracted from pelleted cells using an RNeasy Mini kit (Qiagen), treated with DNase I (Fermentas) and the nuclease was inactivated at 70 °C for 10 min. The cDNA was generated using the ImProm-II Reverse Transcriptase System (Promega) with control reactions lacking reverse transcriptase prepared in parallel. Transcript abundance was determined for selected genes using the oligonucleotide pairs listed in Table S2. Each primer pair was designed to amplify ∼110 bp with *rpoD*, which was used as a reference gene and *zntA* was included to monitor zinc export. Reactions (20 µl) contained 5 ng of cDNA, 400 nM of each primer and PowerUP SYBR Green Master Mix (Thermo Fisher Scientific). Two biological replicates performed in triplicate were analysed using a Rotor-Gene Q 2plex (Qiagen, Rotor-Gene-Q Pure Detection version 2.3.5). Control reactions without a cDNA template were run for each primer pair alongside samples without reverse transcriptase. *C*_q_ values were calculated with LinReg PCR (version 2021.1) after correcting for amplicon efficiency. Change in gene expression relative to the lowest abundance for each transcript was calculated as a series of ΔΔ*C*_q_ values and expressed as a log_2_ fold change in gene expression.

### Determination of cellular metal content

EDTA or DTPMP was added to 50 ml LB broth in 250 ml acid-washed conical flasks prior to inoculation with 1×10^7^ *E. coli* cells. Cultures were grown at 37 °C in an orbital shaker at 130 r.p.m. to inhibit growth by 10–30 % during the mid-log phase (~0.3–0.4 OD_600 nm_, typically 3–4 h of growth). Cell numbers were recorded using a Casy Model TT Cell Counter prior to harvesting. Cells were pelleted by centrifugation (19 000 ***g***, 25 min) and washed three times in 10 ml of 0.5 M sorbitol, 10 mM HEPES pH 7.8. The cell pellet was then digested in 1 ml, 65 % nitric acid (Suprapur, Sigma Aldrich) for a minimum of 16 h. These pellet digests were diluted with 2.5 % nitric acid and 5.89×10^−4^ µM silver standard for ICP (Sigma Aldrich) in a 1 : 8 : 1 ratio. Calibration samples were made using known quantities of metals in nitric acid (ICP multi-element standards, CertiPUR, Sigma Aldrich and Merck) diluted in a matrix-matched solution. Dilutions and a calibration curve were analysed using inductively coupled plasma mass spectrometry (ICP-MS, Thermo XSERIES 2). Instrument control, analysis and quantification were obtained using the software interface PlasmaLab (Thermo Scientific). Mean and standard deviation values were determined from triplicate biological analyses.

## Results

### Isolation of chelant-tolerant mutants by experimental evolution

**Fig. 1.**
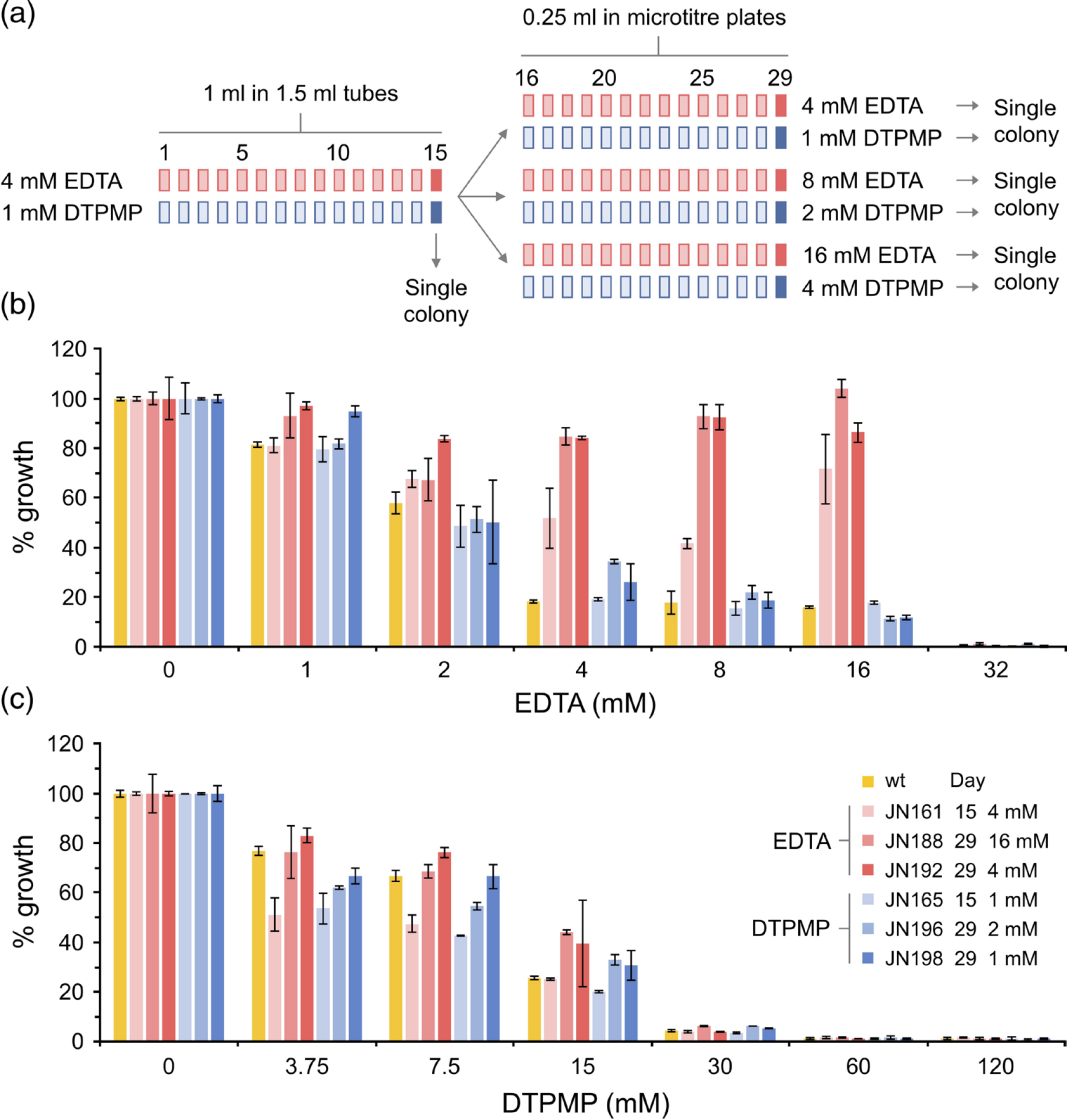
Isolation of *E. coli* chelant-tolerant strains. (**a**) *E. coli* strain BW25113 was iteratively cultured and diluted over 29 days in the presence of sub-MICs of EDTA or DTPMP. Single-colony isolates were recovered at 15 and 29 days. The susceptibility of wild-type and chelant-treated strains to EDTA (**b**) or DTPMP (**c**) was examined. Twofold serial dilutions of each chelant were mixed with each strain and incubated at 37 °C with shaking at 150 r.p.m. for 16 h. Growth was measured at OD_600 nm_ at the end-point and normalized against controls without chelant to give the percentage growth. Mutants selected against EDTA are shown in shades of red, while those exposed to DTPMP are in blue. Results represent the mean and standard deviation of an experiment performed in triplicate. An additional independent repeat produced similar results (Fig. **S1**).

To gain further insight into possible evolvable resistance mechanisms to metal deprivation and the antibacterial action of chelating agents, we attempted to isolate mutants of *E. coli* with resistance to EDTA or DTPMP. These two chelants were selected because of their differential effects on *E. coli* cellular metal content. EDTA depletes cells of manganese with additional reductions in iron and zinc, while DTPMP primarily deprives *E. coli* of iron, resulting in an influx of manganese [16]. *E. coli* K12 (strain BW25113) was cultivated iteratively in sub-MIC levels of each compound over 100–200 generations. The BW25113 strain was chosen, as it is the parent strain for the Keio library of deletion–insertion mutants [30], enabling subsequent comparisons with derivatives from the same genetic background. Bacteria were initially grown in microcentrifuge tubes with daily subculture in either 4 mM EDTA or 1 mM DTPMP (Fig. 1a). After 15 days, cultures were purified to single colonies on agar plates, and a culture from each was stored as a frozen stock. Sub-culturing was continued in 96-well plates, allowing exposure of the bacterial population to higher concentrations of each chelant. After 29 days, these cultures were recovered, and single colonies purified and retained (Fig. 1a). Note that day 15 isolates are not necessarily the direct ancestors of day 29 cells, as single colonies were obtained from a mixed population. A control culture without chelating agents was cultivated under the same conditions over 29 days, yielding strain JN186. Isolates cultivated at the lowest concentration of each chelant at both 15 and 29 days, and an additional higher concentration at day 29, were tested for resistance to EDTA and DTPMP (Fig. 1b, c).

The three strains isolated at low levels of EDTA all showed improved growth in the presence of this chelating agent when compared to the parent strain (Figs 1b and S1a, c). This superior tolerance was readily apparent at higher concentrations of EDTA (4–16 mM), especially with the two strains cultivated for 29 days. In contrast, the DTPMP-exposed strains did not exhibit enhanced tolerance (Figs 1c and S1b, d). None of the EDTA-selected strains had developed cross-resistance to DTPMP. Similarly, all of the strains isolated against DTPMP showed susceptibility to EDTA equivalent to the WT (Figs 1b, c and S1).

### Colony morphology and growth of chelant-selected mutants

The colony morphology of the *E. coli* strains selected against EDTA and DTPMP was examined (Fig. S2). In most cases, there were no marked differences in the colonial appearance of these strains relative to the parental WT, although large and small colony variants were noted with the three EDTA-resistant strains (Fig. S2b–d). The growth of the six chelant-selected isolates was also examined in LB broth without chelant addition (Fig. S3a). All strains, whether isolated against EDTA or DTPMP, displayed a similar slow growth phenotype with a prolonged exponential phase, much slower than the WT. The two strains isolated after 15 days (JN161 and JN165) showed slightly faster growth than those obtained after 29 days (Fig. S3a). A delayed growth phenotype was also apparent with selected strains in larger scale, 50 ml, liquid cultures (Fig. S3b).

### Identification of mutations in chelant-selected strains

**Fig. 2.**
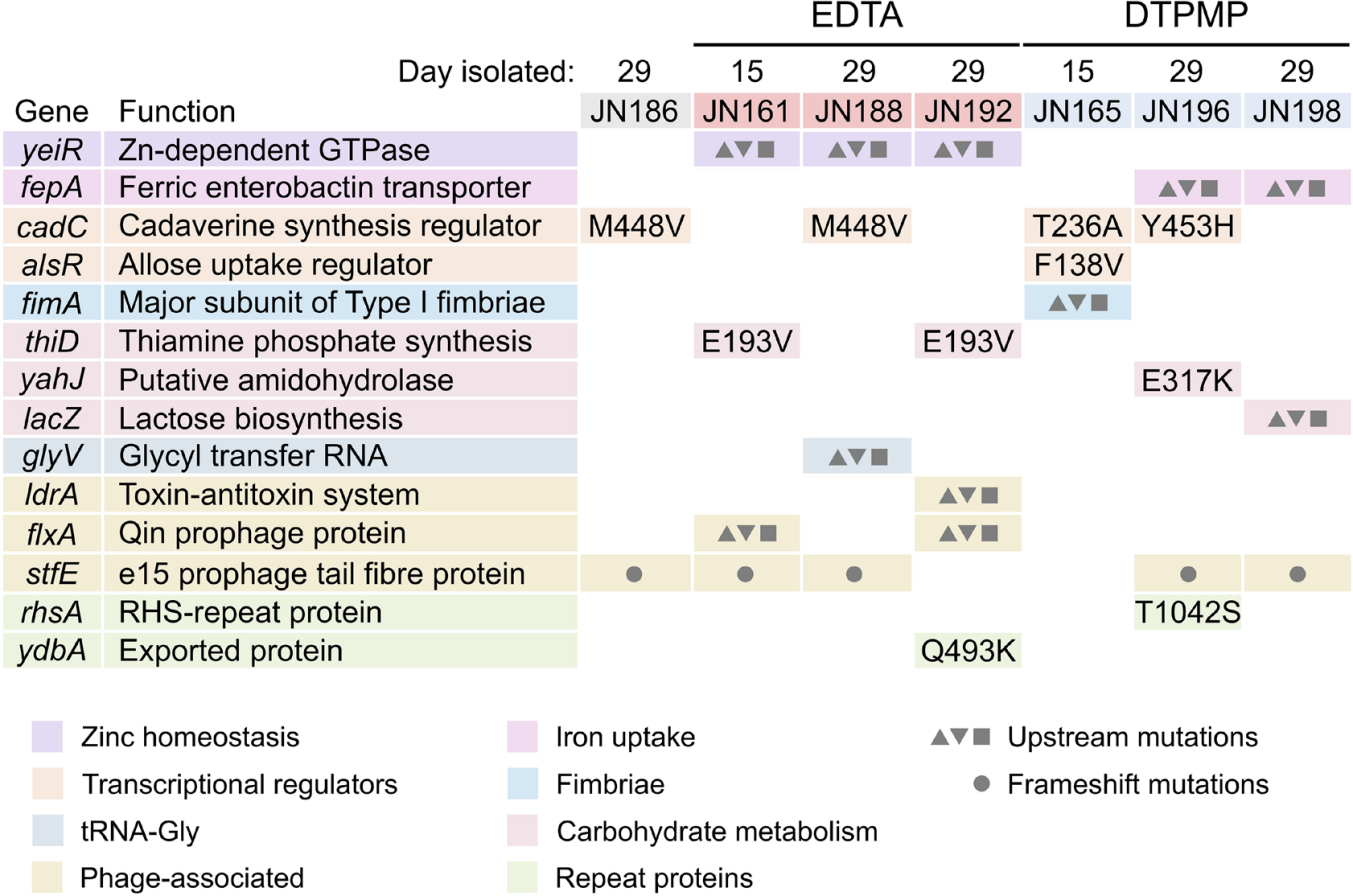
Mutations identified by whole-genome sequencing of *E. coli* chelant-selected strains. Three EDTA- and three DTPMP-exposed isolates and an untreated control (JN186) were selected for sequencing. The genes affected are grouped according to function and specific mutations listed for each strain. Missense mutations that change amino acid sequences of the gene product are shown. JN192 *ydbA* also has an I485M substitution. Gene *stfE* carries several frameshift mutations as the circle symbol indicates. Base changes upstream of genes are indicated by triangular and square symbols reflecting potential upregulation, downregulation or no effect on adjacent gene expression. Synonymous changes affecting *gcvT* (**x1**), *rhsA* (**x4**) and *ydbA* (**x7**) are not listed.

The genome sequences of each chelant-selected strain, including the BW25113 parent and JN186 grown in the absence of EDTA and DTPMP, were obtained. Genomic variants between the parent and derivative strains were identified and are summarized in Fig. 2. Several of the changes are amino acid substitutions that may affect protein functionality, while others lie upstream of genes and may alter transcription levels. None of the mutations were found in every strain, although those upstream of *yeiR* were present in all EDTA-resistant isolates (Fig. 2). The most promising candidates for a role in chelant resistance included a zinc-dependent GTPase (*yeiR*) and the ferric-enterobactin importer involved in iron uptake (*fepA*). Missense mutations in an acid tolerance regulator (*cadC*) were noteworthy as they arose independently in selected EDTA- and DTPMP-exposed strains and also in the untreated control (Fig. 2). Additional mutated genes were not found in all chelant-selected strains and were therefore considered to be less likely to be critical for tolerance (see Fig. S4, which highlights the location of the Glu193Lys substitution in ThiD and provides additional information on these mutations).

### Involvement of *yeiR* in EDTA resistance

**Fig. 3.**
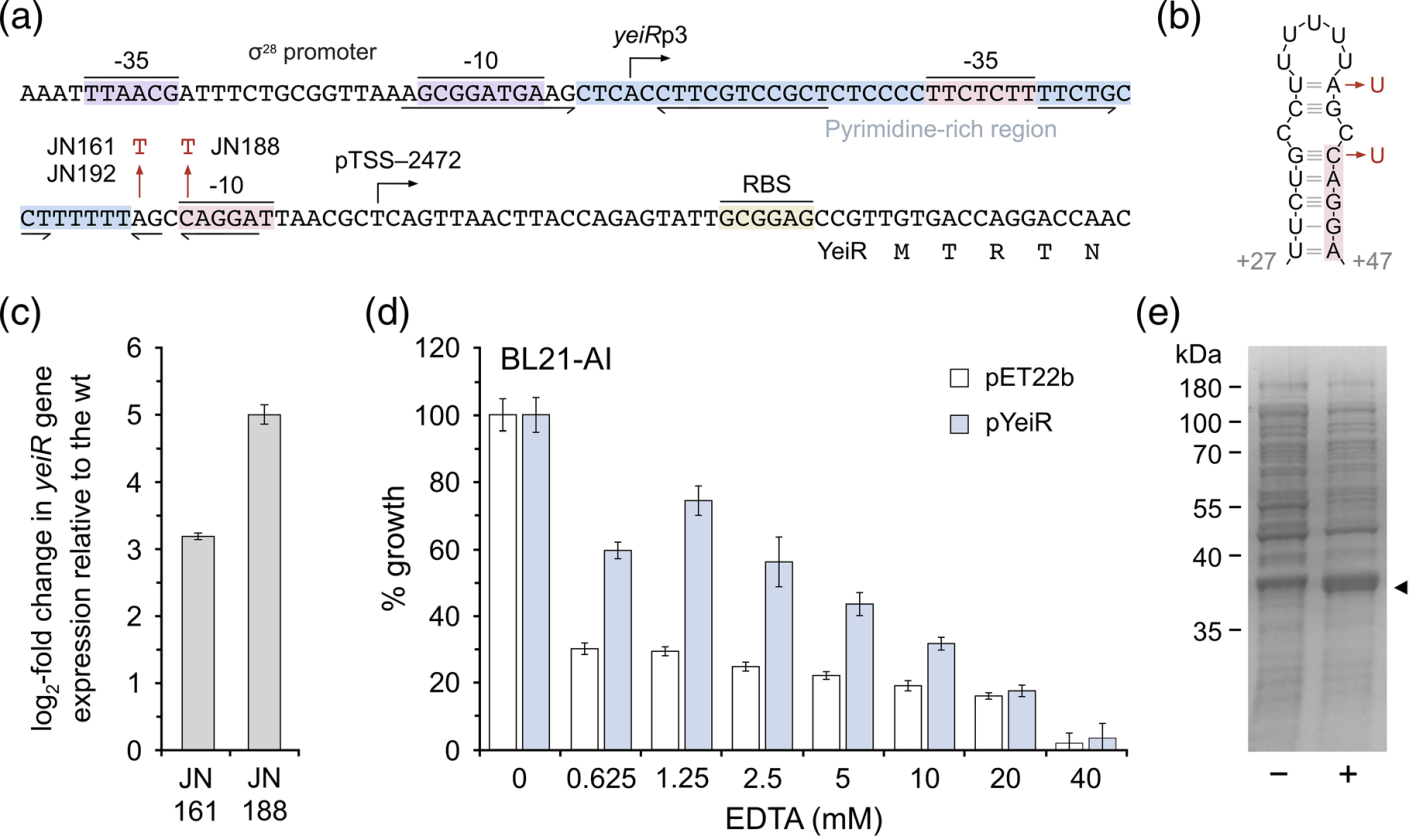
Elevated expression of the YeiR zinc-dependent GTPase is responsible for improved growth in the presence of EDTA. (**a, b**) Location of upstream mutations in the *yeiR* promoter region of EDTA-resistant strains. The −35 and −10 sites (shaded in lilac) of the predicted σ^F^ (σ^28^) promoter, *yeiR*p3; the transcript start site is indicated by a rightward arrow. A second *yeiR* promoter, pTSS-2472 (shaded in red), has been experimentally validated by RNA-Seq, although the sigma factor involved has not been defined. A region of 42 nt contains a high proportion of pyrimidines (90 %; shaded in blue) and overlaps with the pTSS-2472 promoter. A small inverted repeat (underlined) overlaps with the −10 of this promoter and its structure in mRNA and position relative to the *yeiR*p3 transcript start is illustrated (**b**). A longer inverted repeat that could be a regulator binding site overlaps with the −10 of the *yeiR*p3 promoter and is also underlined. Single nucleotide substitutions found in JN161 and JN192 (A to T/U) and JN188 (C to T/U) are highlighted in red (**a, b**). The predicted ribosome binding site (RBS, shaded in yellow) and start of the *yeiR* gene are also shown. (**c**) Mutations in EDTA-selected strains upregulate *yeiR* gene expression. qPCR was used to monitor expression levels of the *yeiR* gene in JN161 and JN188 strains relative to the wild-type; *rpoD* was employed as a reference gene in all samples. Data are the mean and standard deviation of an experiment performed in triplicate. Two additional biological repeats produced similar results (Fig. S5a, b). (**d**) Overexpression of YeiR in *E. coli* promotes resistance to EDTA. BL21-AI cells carrying pYeiR, the *yeiR* gene inserted into pET22b, were grown in LB at 37 °C, expression was induced by the addition of 1 mM IPTG and 0.2 % arabinose, and they were incubated for a further 16 h. BL21-AI carrying the pET22b vector control was run in parallel. Growth was measured at OD_600 nm_ at the end-point and normalized against controls without chelant to give the percentage growth. Results represent the mean and standard deviation of an experiment performed in triplicate. Two additional independent repeats produced similar results (Fig. S5c, d). (**e**) Overexpression of YeiR protein. BL21-AI carrying pYeiR was grown to an OD_600 nm_ of 0.5 and half of the culture was induced as in (**d**) for 3 h before harvesting and lysing cells in an SDS-loading buffer. Total cellular proteins from uninduced (−) and induced (+) cells were separated on 12.5 % SDS-PAGE alongside suitable size markers and the gel was stained with Coomassie blue. The YeiR protein (predicted molecular mass of 36 kDa) in the induced sample is indicated by a black triangle.

In the EDTA-selected strains, two distinct mutations were located in the intergenic region upstream of *yeiR* that could be associated with chelant tolerance (Fig. 3a). An A–T mutation is present at the same location in both JN161 and JN192, while JN188 has a different C–T change nearby; both lie within the experimentally defined pTSS-2472 promoter close to the −10 signal sequence (Fig. 3a). The fact that these mutations must have arisen independently supports the notion that this region contains important regulatory features that are altered favourably for chelant resistance. Interestingly, a short, inverted repeat overlapping the predicted −10 for the pTSS-2472 promoter is predicted to form a short stem–loop in mRNA (Fig. 3b) and both of the isolated mutations would disrupt the stability of this structure. The *E. coli yeiR* gene product is a Zn-dependent G3E (COG0523) family P-loop GTPase involved in zinc homeostasis [37, 38]. Increased expression of YeiR might therefore be expected to help these strains tolerate zinc deprivation in the presence of EDTA. Transcriptional analysis by qPCR confirmed log_2_ three–sevenfold higher levels of *yeiR* gene expression in JN161 and JN188 relative to the WT control (Figs 3c and S5a, b).

To establish whether increased YeiR is directly responsible for EDTA resistance, we inserted the *E. coli yeiR* gene into the pET22b expression vector, placing it under the control of the phage T7 promoter. *E. coli* BL21-AI was transformed with pYeiR and susceptibility to EDTA was assessed following induction of *yeiR* expression with appropriate supplements. Cells carrying the induced pYeiR construct showed substantially improved resistance to EDTA relative to the vector control (Figs 3d and S5c, d). Overexpression of YeiR was confirmed by the detection of high-level expression in the BL21-AI strain (Fig. 3e).

### Screening chelants for metal selectivity using Δ*yeiR* and Δ*yjiA* mutants

**Fig. 4.**
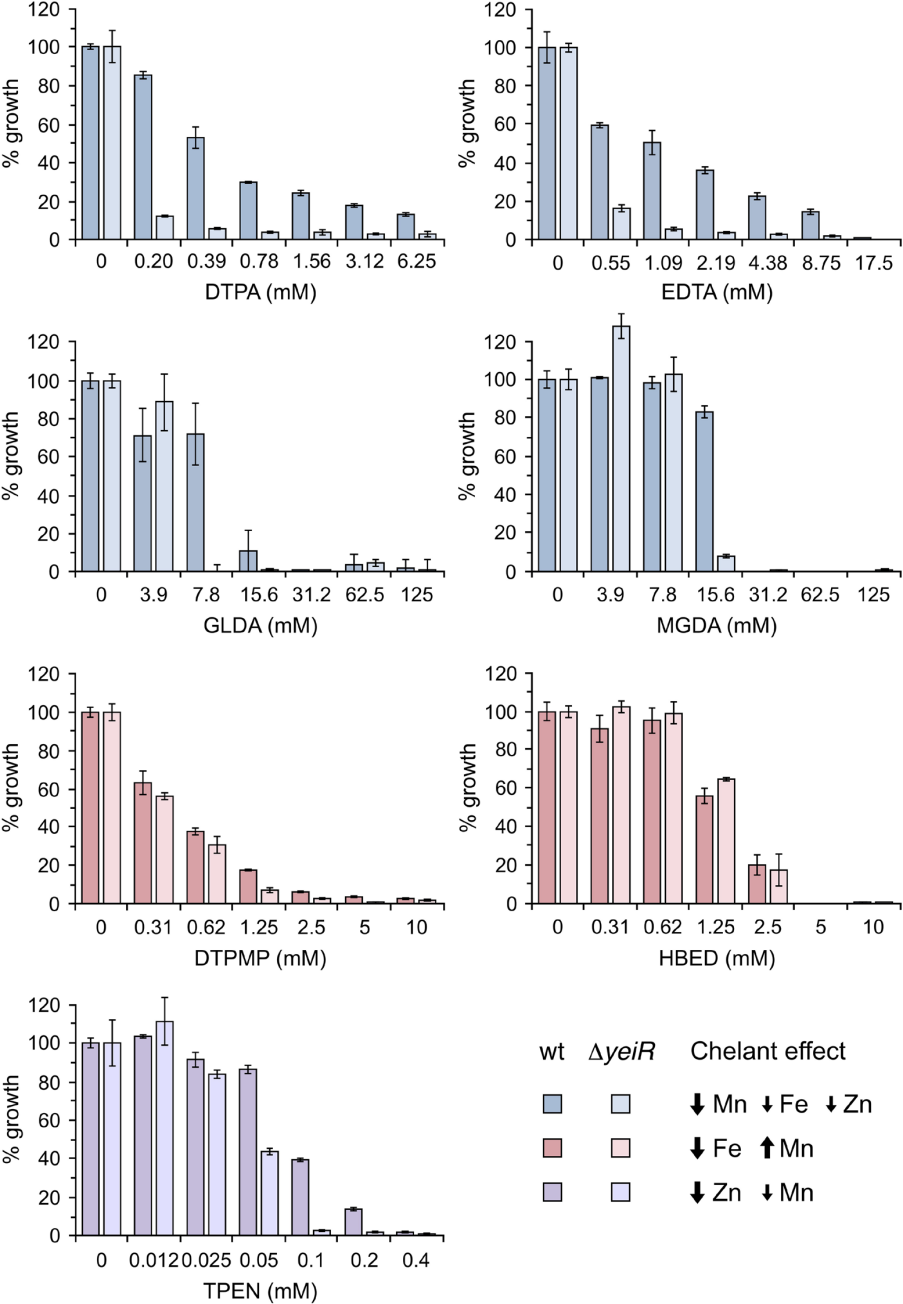
Susceptibility of *E. coli* wild-type (WT) and a *yeiR* mutant to different metal-chelating agents. Diluted cultures of *E. coli* WT (BW25113) and the isogenic Δ*yeiR* mutant were mixed with twofold serial dilutions of each chelant and incubated at 37 °C with shaking at 150 r.p.m. for 16 h. The extent of growth was measured at OD_600 nm_ at the end-point and normalized against controls without chelant to give the percentage growth. Results represent the mean and standard deviation of an experiment performed in triplicate. Two additional independent repeats yielded similar results (Fig. S**6**). Chelant effects on *E. coli* cellular metal content are indicated based on previous ICP-MS analysis [16]. DTPA, EDTA, GLDA and MGDA primarily reduce manganese with additional reductions in iron and zinc (blue). DTPMP and HBED function as iron chelators (red) and TPEN mainly targets zinc (purple).

The YeiR GTPase is highly selective for zinc and strains lacking *yeiR* are known to have increased susceptibility to EDTA [37]. We reasoned that a Δ*yeiR* strain could be used to screen for chelators that affect zinc levels in *E. coli* and so examined the susceptibility of WT and Δ*yeiR* strains to chelating agents with differing chemistries (Table S1). The Δ*yeiR* strain proved highly sensitive to EDTA, as noted previously [37], and also to the related DTPA (Figs 4 and S6). Increased susceptibility of Δ*yeiR* relative to the WT was also apparent with GLDA and MGDA (Figs 4 and S6). Collectively, these four chelants are functionally equivalent in reducing cellular levels of iron, manganese and zinc in *E. coli* [16]. Exposure to TPEN, which has enhanced selectivity for zinc [16, 39], also showed increased sensitivity with the Δ*yeiR* mutant strain. Apart from catechol, no substantial susceptibility was detected with the remainder of the chelates tested (Figs 4, S6 and S7), including DTPMP, PO and HBED, which are known to preferentially deprive *E. coli* cells of iron [16].

A related COG0523 GTPase, YjiA, belongs to a different subset of the family (cluster 3 rather than 7) and is able to bind cobalt, nickel and zinc [40, 41]. We utilized an *E. coli* Δ*yjiA* mutant to screen the same 14 chelants to help identify which metals, if any, might be linked to YjiA activity. However, none of the chelants increased the susceptibility of Δ*yjiA* relative to the *yjiA* ^+^ strain (Figs S8, S9).

### Metal homeostasis in EDTA-resistant mutants

The effect of the *yeiR* promoter mutations on metal homeostasis was initially probed using inductively coupled plasma mass spectrometry (ICP-MS) to determine the cellular levels of six transition metals (Fig. S10). The WT, JN161 and JN188 were exposed to two different concentrations of EDTA, resulting in 10–25 % growth inhibition. In response to EDTA, the WT showed a substantial decrease in manganese levels (13- and 29-fold), along with lesser reductions in iron, zinc and calcium, as noted previously [16]. Similar responses to chelant treatment were apparent with JN161, JN188 and the Δ*yeiR* mutant (Fig. S10). In the absence of EDTA, there was a small, but significant, reduction in levels of iron (*P*<0.01) and zinc (*P*<0.05) in the two EDTA-selected strains compared to the WT (Fig. S10). In contrast, basal levels of manganese, magnesium, calcium and copper were comparable between the strains. Lower cellular concentrations of iron and zinc, and additionally manganese (*P*<0.01), were also found in the Δ*yeiR* strain relative to the WT (Fig. S10).

**Fig. 5.**
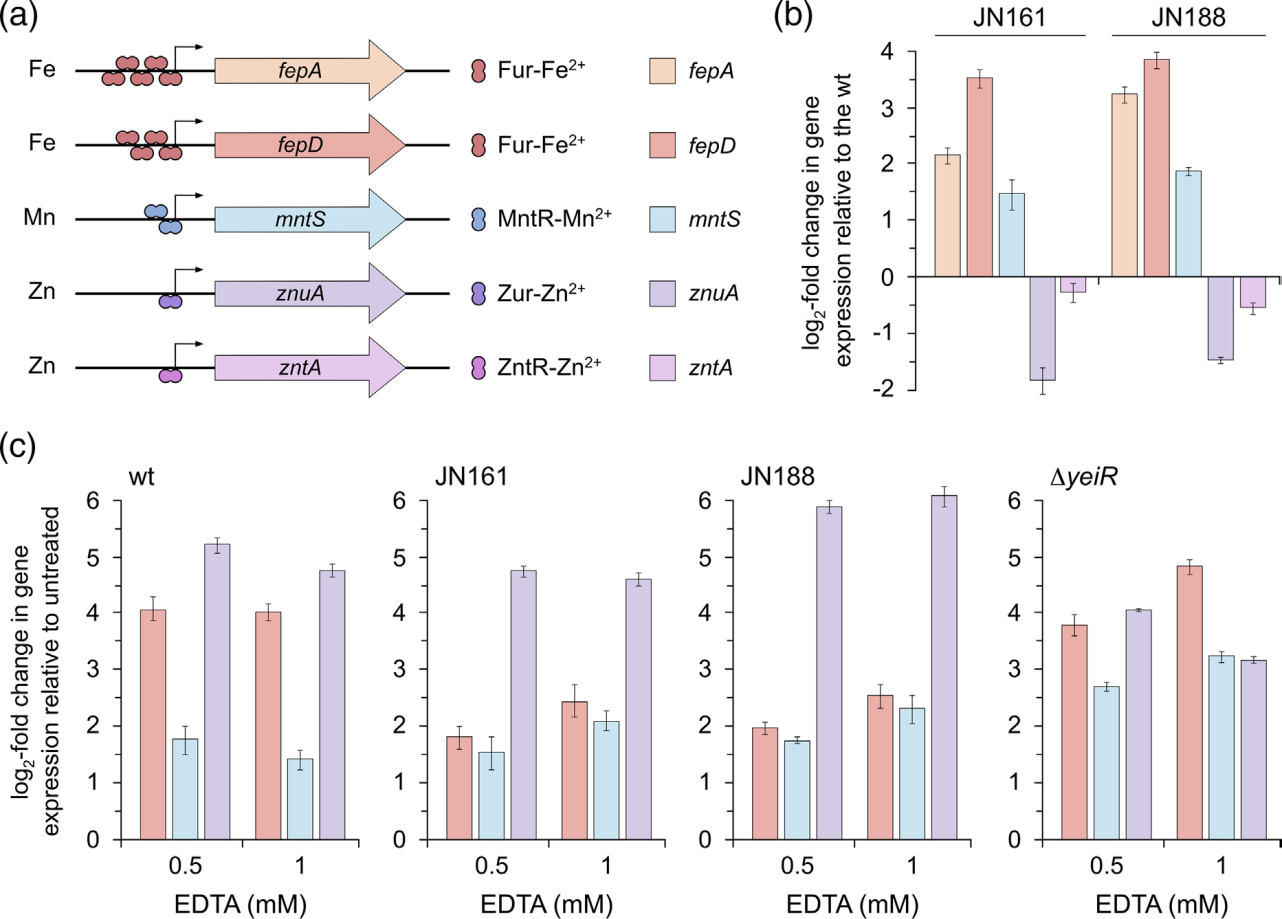
Effect of *yeiR* promoter mutations in EDTA-selected strains on expression of metal-regulated sensors. (**a**) Schematic of genes regulated by appropriate iron, manganese and zinc regulatory pathways selected to monitor cellular responses to chelant treatment. Transcription of *fepA* and *fepD* is negatively regulated by Fur bound to Fe^2+^ [58], *mntS* by MntR bound to Mn^2+^ [72] and *znuA* by Zur bound to Zn^2+^ [73]. All three transcriptional regulators are repressors that limit expression under metal-replete conditions. ZntR-Zn^2+^ activates expression of *zntA*, the zinc exporter [74]. Promoters are indicated by arrows, as are the number of binding sites for each regulator. Genes are coloured to match the data in (b, c). (**b**) Relative metal sensor gene expression between mutants and WT in the absence of chelant. qPCR was used to monitor expression levels of each metal-regulated gene in strains JN161 and JN188 relative to the WT. (**c**) Relative metal sensor gene expression between mutants and WT in the presence of EDTA. qPCR was used to monitor expression levels of *fepD*, *mntS* and *znuA* in the BW25113 WT, JN161, JN188 and JW2161 (Δ*yeiR::kan*) exposed to either 0.5 or 1 mM EDTA relative to untreated controls. *rpoD* was employed as a reference gene in all samples. Data in (b, c) are the mean and standard deviation of an experiment performed in triplicate; an additional biological repeat produced similar results (Fig. S11b, c).

To investigate this further we examined the expression of genes whose transcription is regulated in response to metal starvation. ICP-MS measurements cannot distinguish between metals sequestered by chelators externally, at the cell surface or in the cytosol, whereas monitoring gene expression provides a direct readout of the bacterial response to intracellular metal levels. Attention focused on genes induced in response to iron (*fepD*), manganese (*mntS*) or zinc (*znuA*) limitation. Gene expression of *zntA*, encoding a Zn^2+^ exporter, was also employed for comparisons between the WT, JN161 and JN188 (Fig. 5a). Expression was monitored by qPCR following exposure to EDTA at concentrations that restricted *E. coli* growth by 10–25 %. Oligonucleotides (Table S2) and their respective gene targets have been validated previously with EDTA using *rpoD* as a reference [36]. Time course experiments with 1 mM EDTA showed differential responses in *fepD*, *mntS* and *znuA* transcription (Fig. S11a). An initial rapid starvation of manganese was indicated by increased *mntS* expression at 10 min, which tails off rapidly, followed by higher and more sustained *fepD* and *znuA* responses, consistent with iron and zinc restriction by EDTA (Fig. S11a). Since 30 min was optimal for measuring of the expression of the three genes, all further experiments were conducted using this time point. In the absence of EDTA, elevated expression of *fepD* and, to a lesser extent, *mntS* was evident in the JN161 and JN188 mutants relative to the WT (Figs 5b and S11b). The results suggest that the increased levels of YeiR in mutant strains cause some reduction in iron availability, even in the absence of chelant. An iron starvation response in the mutant strains was verified using *fepA* (Figs 5b and S11b). Expression of *znuA* was reduced relative to the WT in JN161 and JN181, suggesting that zinc levels are sufficient in the mutants; additionally, no significant zinc export via elevated *zntA* expression was detected (Figs 5b and S11b).

Next, the cellular response of the sensor genes to EDTA in WT and mutants was compared (Figs 5c and S11c). A similar pattern of responses was observed with *mntS* and *znuA* expression, however, while a fourfold (log_2_) increased expression of *fepD* was seen in the WT, this was only ~twofold in the JN161 and JN181 mutants (Fig. 5c). The increased basal levels of *fepD* in the absence of chelant (Fig. 5b) probably account for the reduced response in these strains. In the Δ*yeiR* mutant, the expression levels of the three genes were broadly comparable with those of the WT when exposed to EDTA (Figs 5c and S11c).

### DTPMP-selected mutants and elevated expression of *fepA*

**Fig. 6.**
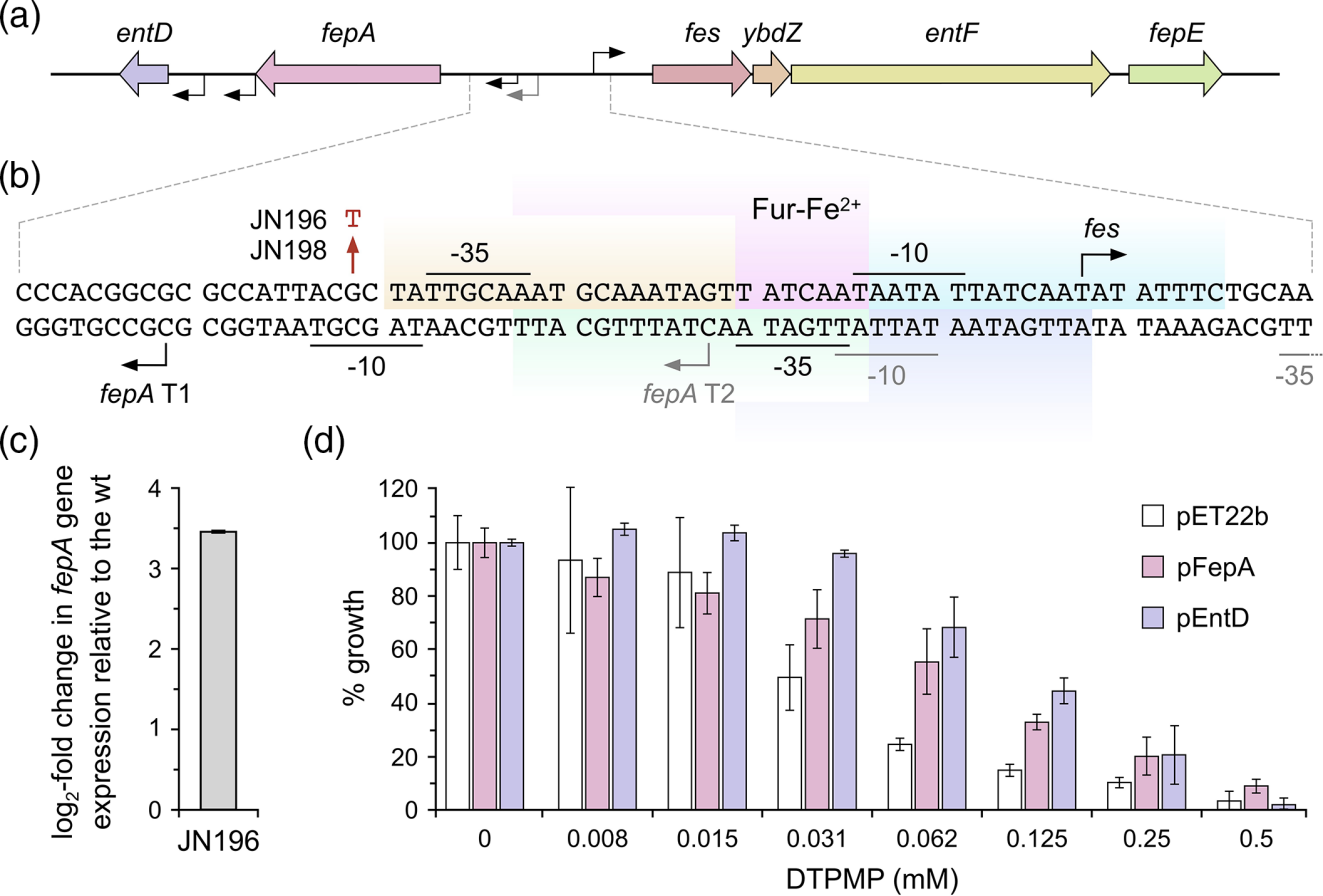
Elevated expression of *fepA* or *entB* improves growth in the presence of DTPMP. (**a**) The promoter region of the divergently transcribed *fepA-entD* and *fes-ybdZ-entF-fepE* operons involved in enterobactin biosynthesis and Fe^3+^-enterobactin uptake. Arrows indicate the orientation of the promoters for each set of genes. (**b**) Sequence of the *fepA* and *fes* promoter region and location of Fur binding sites. The −10 and −35 sites indicate the differently orientated promoters for *fepA* and *fes*. Transcript start sites are indicated by arrows, including the minor (**t1**) and major (**t2**) transcripts for *fepA*; the latter signals are distinguished in grey [44, 75]. Five overlapping binding sites for the Fur–Fe^2+^ repressor [58] are indicated by shaded areas in different colours. The single-nucleotide substitution (G to T) found in JN196 and JN198 is highlighted in red. Additional Fnr, H-NS and Crp binding sites are not shown. (**c**) The promoter mutation in a DTPMP-selected strain upregulates *fepA* gene expression. qPCR was used to monitor expression levels of the *fepA* gene in JN196 strains relative to WT; *rpoD* was employed as a reference gene. Data are the mean and standard deviation of an experiment performed in triplicate. A separate independent repeat produced similar results (Fig. S12a). (**d**) Overexpression of FepA or EntD proteins in *E. coli *. BL21-AI cells carrying pFepA or pEntD, the *fepA* or *entD* genes, respectively, inserted into pET22b, were grown in LB broth at 37 °C, expression was induced by addition of 0.5 mM IPTG and 0.1 % arabinose, and they were incubated for a further 16 h. BL21-AI carrying the pET22b vector control was run in parallel. Growth was measured at OD_600 nm_ at the end-point and normalized against controls without chelant to give the percentage growth. The results represent the mean and standard deviation of an experiment performed in triplicate. Two additional independent repeats produced similar results (Fig. S12b, c).

Of the three DTPMP-selected strains, JN196 and JN198, both isolated at 29 days (Fig. 1), carry an identical mutation in the intergenic region between the divergently transcribed *fepA-entD* and *fes-ybdZ-entF-fepE* operons (Fig. 6a). Both operons participate in enterobactin biosynthesis and Fe(III)-enterobactin import [42]. The G–T transversion in the *fepA* promoter region in mutant strains affects the −10 sequence of the weaker of the two *fepA* promoters (Fig. 6b) and appears to produce a more favourable σ^70^ RNA polymerase binding site (5′-TAGAGT-3′). Upregulation of FepA, the ferric-enterobactin uptake channel [43, 44], might be expected to improve iron retrieval in the presence of low levels of DTPMP during selection. Transcription of the *fepA* gene in one of the mutants, JN196, was analysed by qPCR in the absence of DTPMP and confirmed that *fepA* has three–fourfold (log_2_) improved expression relative to its parent strain (Figs 6c and S12a).

To investigate whether increased FepA levels can help combat chelant-mediated iron limitation, the *fepA* gene was inserted into pET22b, which permits its controlled expression in appropriate strains. The *entD* gene, encoding enterobactin synthase component D, was also cloned, as it lies downstream of *fepA* and its expression could be elevated despite possessing its own promoters (Fig. 6a). The two constructs along with the vector control were introduced into the *E. coli* strain BL21-AI and their effects on DTPMP resistance investigated (Fig. 6d). Growth improvement with constructs expressing either FepA or EntD relative to the vector control was evident in induced cells exposed to 0.031–0.25 mM DTPMP (Figs 6d and S12b, c).

The cellular metal content of the WT and JN196 was compared (Fig. S13a). Similar quantities of each of the six trace metals were detected in each strain in the absence of DTPMP, and both showed equivalent cellular responses to chelant exposure (Fig. S13a), with reduced iron and elevated manganese, as reported previously [16]. At higher concentrations of DTPMP, reductions in iron were more pronounced, and manganese and zinc levels were also lowered, but WT and mutant still showed comparable responses to chelant treatment (Fig. S13b).

As with the EDTA strains, qPCR was employed to monitor the expression of *fepD*, *mntS* and *znuA* in JN196 relative to the WT strain in the absence of chelant (Fig. S14). The mutant showed elevated basal levels of the *fepD* and *mntS* genes, but not *znuA*, indicating that the strain is experiencing deficiencies in the availability of iron and manganese.

### CadC mutations

**Fig. 7.**
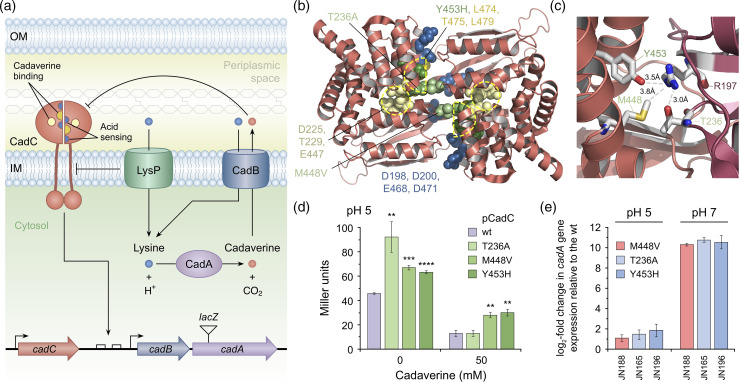
Mutations in the CadC protein result in constitutive expression of the *cadAB* operon. (**a**) The CadCAB acid tolerance system. In response to acid stress (pH <6.8) and in the presence of lysine, the CadC transcriptional activator induces the expression of the *cadBA* operon. *cadB* encodes a lysine/cadaverine antiporter and *cadA* a lysine decarboxylase; the latter combines lysine and a proton to generate the more alkaline cadaverine and carbon dioxide to increase the intracellular pH. CadB imports lysine to the cytosol and exports cadaverine to the periplasmic space, where it helps increase the extracellular pH. LysP is a lysine-specific importer and a co-sensor and inhibitor of CadC. Exported cadaverine acts as a feedback inhibitor of CadC activation. Acid sensing and cadaverine binding occur at the interface between CadC subunits in the periplasm. Five negatively charged residues in CadC along the dimer surface are essential for activation of CadC at low pH, most likely by protonation that reduces charge repulsion and allows conformational changes to be transmitted that trigger binding to the *cadBA* promoter inside the cell [33, 46, 76]. The *cadC* gene is located immediately upstream of the *cadBA* operon and two CadC binding sites are located within the *cadBA* promoter (the *cadC* and *cadBA* promoters are indicated by arrows). The location of the *cadA′*∷*lacZ* insertion used to monitor *cadAB* expression in (**d**) is indicated. Adapted from Brameyer *et al.* [77] and Jung *et al.* [45]. (**b**) Structure of the periplasmic domain of *E. coli* CadC dimer (Protein Data Bank: 3LY7 [47]), highlighting the location of mutated residues. The dimer is shown from above looking towards the cytoplasmic membrane with the dimer interface positioned from top to bottom. Residues mutated in chelant-selected and control strains are highlighted in green (T236A in JN165, M448V in JN186/188 and Y453H in JN196). Negatively charged residues at the dimer interface important in detecting low pH are coloured blue (D198, D200, E461, E468 and D471 [76]). Residues involved in cadaverine binding are coloured yellow (encircled by dashed yellow lines) and are clustered at the dimer interface (Y453, L474, T475 and L479) and central cavity (D225, T229 and E447) sites. (**c**) Dimer interface contacts involving CadC mutated residues. Tyr463 and Thr236 form hydrogen bonds with Arg197, while Met448 makes hydrophobic contact with this residue. The Thr236Ala, Ty463His and Met448Val mutants would all disrupt this network of contacts. The two CadC subunits are coloured different shades of red, on the left and right, at the dimer interface. Protein structures were generated in Pymol. (**d**) Effect of CadC mutations on *cadBA* expression in the presence or absence of cadaverine. Reporter gene assays were conducted with *E. coli* EP314 (*cadC1*∷Tn*10 cadA′*∷*lacZ*) complemented with plasmid-encoded CadC (wild-type, WT) or the indicated CadC mutants. An overnight culture grown in LB at pH 7 was shifted to LB at pH 5 in the presence or absence of 50 mM cadaverine. Cells were harvested after 3 h of growth (mid-log) and β-galactosidase activity was measured. The percentage of residual activity was calculated in relation to the same condition without cadaverine. Results are the mean and standard deviation of three independent experiments, the *t*-test was used to compare the pCadC WT with each mutant; **P<0.01, ***P<0.001 and ****P<0.0001 (*n*=3). (**e**) Expression of *cadA* in chelant-selected strains carrying mutations in *cadC*. qPCR was used to monitor expression levels of the *cadA* gene in JN165, JN188 and JN196 relative to the WT; *rpoD* was employed as a reference gene. Data are the mean and standard deviation of an experiment performed in triplicate. An additional biological repeat produced similar results (Fig. **S15**).

The three unique amino acid substitution mutations found in CadC were the most intriguing of the changes identified in the screen for chelant-resistant mutations (Fig. 2). CadC is a sensor and regulator of acid stress that is bound to the cytoplasmic membrane and has a periplasmic domain responsible for signalling low pH in association with LysP (Fig. 7a) [45]. When cells are exposed to low pH in the presence of lysine, the inhibitory interaction between LysP and CadC is weakened, rendering CadC susceptible to protonation and capable of activating expression of the *cadBA* operon. *cadB* encodes a lysine/cadaverine antiporter and *cadA* is a lysine decarboxylase that converts lysine, by proton addition, to the more alkaline cadaverine to increase both intracellular and extracellular pH (Fig. 7a) [46].

The three CadC substitutions (Fig. 2) found in JN165 (Thr236Ala), JN188 (Met448Val) and JN196 (Tyr453His) appear to have arisen independently in EDTA- or DTPMP-treated cells. Significantly, JN186, which was not exposed to either chelating agent, carries an identical mutation to JN188 (Fig. 2). Strikingly, the mutations cluster in the C-terminal periplasmic sensor domain of CadC at the dimer interface (Fig. 7b, c) and all three are involved in inter-subunit interactions [47]. In fact, Tyr236 and Met448 from one subunit, together with Thr236 from the other, form a network of contacts with another conserved residue, Arg197, that would be disrupted by any of the three substitution mutations (Fig. 7c).

### The *cadC* mutations result in constitutive expression of the *cadBA* operon

As in previous work [33], a *cadC1*∷Tn*10 cadA′*∷*lacZ* strain (EP314) carrying constructs expressing the *cadC* WT and mutant genes was employed to monitor effects on *cadAB* activation (Fig. 7d). The WT *cadC* gene was inserted into pET22b and the three substitution mutations were introduced in this construct by site-directed mutagenesis and these were examined alongside negative (empty vector) and positive (pCadC WT) controls. EP314 lacks T7 RNA polymerase and so expression of the cloned genes depends on fortuitous expression, although this is more likely given the high copy number of the vector. Gene expression from the *cadBA* operon was measured by β-galactosidase activity after switching cells from growth at pH 7 to pH 5 in the presence of additional lysine without the feedback inhibitor cadaverine. All three of the mutant CadC constructs confer improved expression of the *cadBA* operon relative to the WT (Fig. 7d). Addition of cadaverine reduced the expression of *cadBA* in the WT and the three mutants. However, the relative reduction in the absence or presence of cadaverine differed, with a 3.5-fold reduction in the CadC WT and a greater reduction (7.4-fold) in T236A due to the improved expression of *cadBA* seen in the latter without cadaverine addition (Fig. 7d). Although the M448V and Y453H mutants showed improved *cadBA* expression in the presence of cadaverine, there is less of a reduction relative to the CadC WT in treated versus untreated conditions (approximately twofold with each) (Fig. 7d). The results suggest that there is some influence on cadaverine binding by the CadC mutations that have arisen in these strains, although the effects are small.

To provide a more direct assessment of the impact of *cadC* mutations on *cadBA* expression, we used qPCR to monitor *cadA* expression in the relevant chelant-selected strains (Figs 7e and S15). At pH 5, the three strains showed similar levels of expression as the WT under conditions where CadC would be expected to activate expression of *cadBA* for acid tolerance (Figs 7e and S15). In contrast, at pH 7 all three mutants were fully activated in *cadA* expression. This can be seen most clearly in the *C*_q_ values for each of these strains under these two conditions, showing that *cadC* ^+^ strains activate *cadA* expression at pH 5 relative to pH 7, whereas all mutants are activated to the same high levels at both pH 5 and 7 (Fig. S16).

### CadC mutations help maintain a neutral pH during bacterial growth

**Fig. 8.**
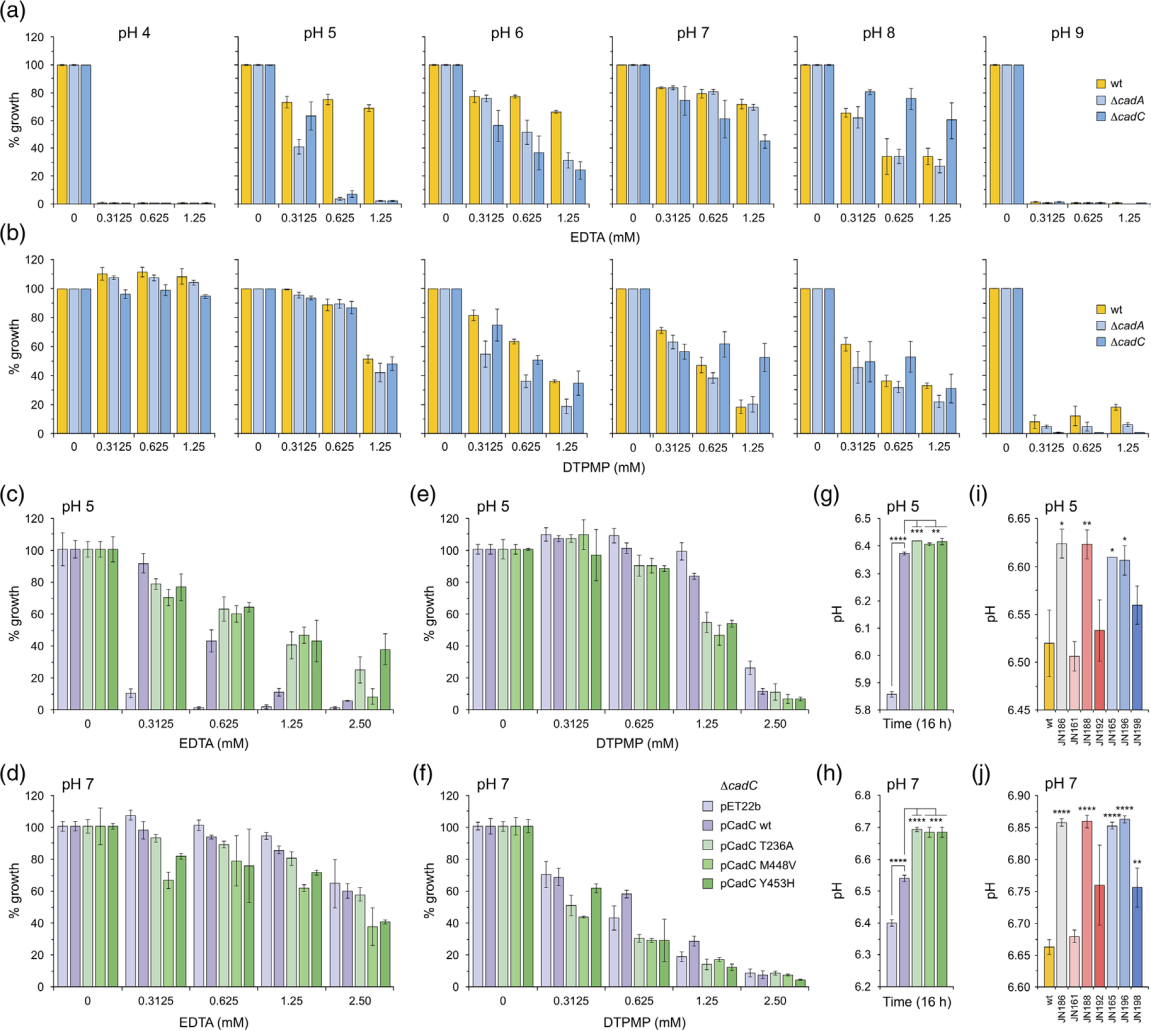
Effect of CadC mutations on *E. coli* susceptibility to chelants under acidic conditions. (**a, b**) Susceptibility of *E. coli* WT, *cadA* and *cadC* mutants to EDTA (**a**) and DTPMP (**b**) under acidic and alkaline conditions. *E. coli* WT, Δ*cadA* and Δ*cadC* mutants were mixed with serial dilutions of each chelant in LB at the indicated pH and incubated at 37 °C with shaking at 150 r.p.m. for 16 h. The extent of growth was measured at OD_600 nm_ at the end-point and normalized against controls without chelant to give the percentage growth. Results represent the mean and standard deviation of an experiment performed in triplicate. Two additional independent repeats yielded similar results (Fig. **S17**). (**c–f**) Effect of plasmid-encoded CadC mutants on *cadC* mutant susceptibility to EDTA (**c, d**) and DTPMP (**e, f**) at pH 5 and 7. The Δ*cadC* mutant transformed with complementing plasmids expressing the CadC WT, mutant variants or the vector control (pET22b) were tested for chelant susceptibility in LB at each pH and incubated and analysed as in (**a**). (**g, h**) Effect of plasmid-encoded CadC mutants on the pH of LB media after bacterial cultivation. Overnight cultures (5 ml) of the Δ*cadC* mutant transformed with complementing CadC plasmids were prepared in LB pH 5 (**g**) or pH 7 (**h**). After incubation at 37 °C with shaking at 150 r.p.m. for 16 h, cells were pelleted by centrifugation and the pH of the media was recorded. The results are the mean and standard deviation of three independent experiments. (**i, j**) Effect of chelant-isolated and control strains on the pH of LB media after bacterial cultivation. Overnight cultures of the wild-type and the EDTA- and DTPMP-isolated strains were prepared in LB pH 5 (**i**) or pH 7 (**i**). After incubation at 37 °C with shaking at 150 r.p.m. for 16 h, cells were pelleted by centrifugation and the pH of the media was recorded as described in (g, h). The results are the mean and standard deviation of three independent experiments. In (g–j), the *t*-test was used to compare WT and mutants, or other controls, as indicated; *P<0.05, **P<0.01, ***P<0.001 and ****P<0.0001 (*n*=3).

To assess potential links between *cadC* mutation, *cadBA* expression and chelant tolerance, we examined the susceptibility of Δ*cadA* and Δ*cadC* mutants to EDTA and DTPMP under pH conditions that span the range that *E. coli* normally encounters in the human gastrointestinal tract [48, 49]. The *E. coli* WT proved hypersensitive to EDTA at pH 4 and 9 and less so between pH 5 and 7 (Figs 8a and S17a). In contrast, the Δ*cadA* and Δ*cadC* mutants proved more sensitive than the WT at pH 5 and to a lesser extent at pH 6, highlighting an elevated toxicity of EDTA when this acid tolerance pathway is unavailable. The effect of DTPMP on *E. coli* at each pH was distinctly different, with comparable sensitivities of WT, Δ*cadA* and Δ*cadC* mutants across the range (Figs 8b and S17b). Increased susceptibility to DTPMP was evident at higher pH, consistent with improved metal binding as the chelant becomes deprotonated.

The CadC mutant constructs in pET22b were assessed for their capacity to promote resistance to EDTA or DTPMP in a Δ*cadC* mutant strain at pH 5 and pH 7 (Fig. 8c–f). Cells carrying the vector control were extremely sensitive to EDTA at pH 5, while resistance was substantially improved with the pCadC WT construct (Figs 8c and S18a, b). The pCadC mutant variants displayed a greater level of resistance than the pCadC WT at higher concentrations of EDTA (0.625–2.5 mM; Figs 8c and S18a, b). There was no similar growth improvement with EDTA between the plasmids at pH 7 (Fig. 8d), nor was there much difference with exposure to DTPMP at either pH 5 or pH 7 (Fig. 8e, f).

Given that Δ*cadA* and Δ*cadC* strains show increased susceptibility to EDTA under mildly acidic conditions (Fig. 8a), we examined the pH of the media following overnight growth of the Δ*cadC* mutant carrying the pCadC plasmids at either pH 5 (Fig. 8g) or pH 7 (Fig. 8h). The pH of the LB media rose from the starting point at pH 5 in Δ*cadC* cells carrying pET22b to 5.85 and further increased with pCadC WT (Fig. 7g). Although differences were small, there was a further improvement towards more physiological conditions with the pCadC mutants (Fig. 8g; *P*<0.01). In contrast, bacteria grown in LB at pH 7 produced a more acidic pH of 6.4 with the vector control and less so (6.55) with the pCadC WT (Fig. 8h). An even smaller reduction in pH was apparent with the pCadC variants (Fig. 8h; *P*<0.001), in keeping with constitutive expression of *cadAB* that will elevate intracellular and extracellular pH. Since these differences were evident in a Δ*cadC* mutant background complemented by CadC plasmids, we used the same approach to examine the effects of the originally isolated chelant-selected strains on media pH. The pattern of results was similar, with those strains that carry a CadC mutation raising the media pH more effectively at pH 5 (Fig. 8i; *P*<0.05) and reducing it to a lesser extent when pH 7 was the starting condition (Fig. 8j; *P*<0.01). The CadC mutations could potentially improve growth of *E. coli* in the presence of chelants by avoiding acidic conditions, however, neither EDTA nor DTPMP exhibit increased toxicity under mildly acidic conditions with the WT (Fig. 8a, b). Moreover, no improved chelant tolerance was evident with the CadC M448V mutant strain, JN186, at either pH 5 or pH 7 (Fig. S19), despite its capacity to effectively neutralize the growth medium (Fig. 8i, j).

### Susceptibility of *E. coli cadC* mutants to carvacrol and triclosan

Mutations in the C-terminal periplasmic sensing domain of CadC have been isolated previously following the cultivation of *E. coli* at sub-MIC levels of two membrane-active antimicrobials, triclosan [50] and carvacrol [51]. CadC A220T, M322T, R333S, N466Y and D506E mutations arose rapidly in experimental evolution with *E. coli* exposed to triclosan [50]. *E. coli* O23:H52 carvacrol-resistant strains were also isolated that carried a CadC Y504H substitution [51], a residue located at the dimer interface [47]. To determine whether the *cadC* mutants identified in our selective screen could confer resistance to either of these compounds, the relevant mutant strains were assessed for susceptibility to carvacrol (Fig. S20a) and triclosan (Fig. S20b). None of the *cadC* mutant strains displayed any improved tolerance of these antibacterial agents at pH 5 or 7.

## Discussion

Bacteria regularly encounter challenges in retrieving vital metal ions from their surroundings. Such hindrances can arise simply through competition for resources from other species in the microbial community, each with the capacity to export and import diverse metallophores [13, 14]. However, for pathogenic species, the vertebrate host purposefully restricts metal availability to curb microbial proliferation and even, occasionally, as a precursor to eradication within macrophages [1]. While much information has been garnered on the processes of nutritional immunity and bacterial counter strategies, comparatively little is known concerning how bacteria might evolve under the selective pressure of metal limitation [52–54]. In this study, we harnessed the metal sequestering properties of two chelating agents to probe how tolerance develops through alterations in the bacterial genome. This experimental approach also mirrors the scenario whereby resistance might emerge following the deployment of chelating agents or siderophores as antibacterial agents. Our experiments with EDTA and DTPMP reveal how mutations arise that upregulate a zinc delivery pathway, via YeiR, and the Fe(III)-enterobactin biosynthesis and import system, via FepA and EntD, that contribute to improved tolerance of metal restrictive conditions. Our study also reveals how changes in pH can influence metal chelant toxicity and the apparent selective advantage of maintaining neutral pH conditions for *E. coli* during experimental evolution in unbuffered media.

All three mutants isolated in the presence of EDTA carried a mutation upstream of the *yeiR* gene, encoding a zinc-dependent GTPase with a predicted role in zinc homeostasis [37, 38]. Since *yeiR* mutants are susceptible to EDTA [37, 55], it seemed plausible that the two distinct mutations found here would lead to increased expression of the *yeiR* gene and aid in management of zinc restriction [16]. YeiR belongs to the COG0523 subset of the G3E GTPase superfamily that appear to be metal chaperones associated with targeted cobalt, iron and zinc delivery [40, 56, 57]. YeiR is highly selective for zinc [37, 38] and likely fulfils a critical role in distributing zinc to essential, as yet undefined, metalloenzymes under limiting conditions, such as exposure to EDTA. In two of the three EDTA-resistant strains, elevated *yeiR* transcription was confirmed by qPCR and, significantly, overproduction of YeiR alone was sufficient to improve *E. coli* growth in the presence of EDTA. Unexpectedly, increased expression of *yeiR* in mutant strains caused iron deprivation, as judged by upregulation of the Fur–Fe^2+^-regulated *fepA* and *fepD* genes [43, 44, 58]. Most likely this arises by mismetallation of excess YeiR with iron. It is possible that improved zinc delivery combined with a pre-activated ferric-enterobactin response aids in EDTA tolerance. Measurement of metals associated with the cell revealed that EDTA-resistant strains contain less iron and zinc, despite upregulation of pathways for their improved homeostasis. It is possible that lower levels of zinc can be managed more efficiently by the increased YeiR chaperone availability and compensatory reductions in iron may have beneficial effects in limiting destructive hydroxyl radical generation [59].

The hypersensitivity of *yeiR* mutants to EDTA [37] suggested that a Δ*yeiR* strain could be employed to screen for chelating agents that induce zinc starvation. In addition to EDTA, four chelating agents, DTPA, GLDA, MGDA and TPEN, known to affect cellular zinc levels [16] did preferentially inhibit the growth of the Δ*yeiR* mutant. In contrast, the Δ*yeiR* strain showed no substantially increased susceptibility to all other chelators tested, including several known iron-selective chelants, suggesting that none affect zinc levels. YjiA encodes a COG0523 GTPase related to YeiR that appears to share a preference for zinc [38, 41, 60] and so a Δ*yjiA* strain was also screened against all 14 chelants. In contrast to Δ*yeiR*, the Δ*yjiA* mutant showed no difference in susceptibility relative to the WT for any of the chelants tested. This could indicate that YjiA is not involved in the delivery of iron, manganese or zinc targeted by these chelants but instead targets metals such as cobalt or nickel. Alternatively, *E. coli* growth may not be affected by the targeted metal delivery provided by YjiA or it may be involved in cellular processes unrelated to metal delivery. YjiA is implicated in the carbon starvation response [41] and contributory metalloenzymes may not be crucial under the conditions employed here.

In contrast to the EDTA-resistant strains, the DTPMP isolates showed the same level of chelant susceptibility as the BW25113 parent. However, a mutation found in both strains isolated after 29 days exposure to DTPMP altered one of the promoters of the Fur–Fe^2+^-regulated *fepA-entD* operon, improving levels of downstream transcription. EntD fulfils an essential role in enterobactin biosynthesis, a siderophore produced by *E. coli* for iron sequestration [42]. Exported enterobactin complexed with iron is then recovered by the integral outer-membrane receptor FepA before delivery to FepBCDG and import to the cytosol [61]. Cells deficient in components of this iron uptake pathway are significantly more sensitive to DTPMP [16]. Overexpression *in trans* of either *fepA* or *entD* improved *E. coli* growth against low concentrations of DTPMP, suggesting that augmented iron uptake contributes to chelant tolerance. Presumably, the threefold increase in *fepA-entD* expression in DTPMP-selected strains provides some improvement in chelant tolerance but the growth inhibition assay lacks the sensitivity to detect such a benefit. No major changes in cellular metal content were observed in analysis of a representative DTPMP isolate relative to its parent strain (Fig. S13). However, some imbalance in metal levels was detected by qPCR of sensor genes, indicating that the mutant bacteria are experiencing iron and manganese insufficiency even in the absence of chelant (Fig. S14). Secondary disruption of metal homeostasis was also found with *yeiR* upregulation in EDTA-selected strains and suggests that despite potential benefits in tolerating low zinc and iron restriction, there are significant perturbations in the normal functionality of metal homeostasis.

Three unique mutations were isolated in the *cadC* gene whose product serves as an activator of the *cadBA* operon in response to acid conditions [45, 46]. Significantly, the three mutations found in the chelant-adapted strains cluster together at the dimer interface and include Tyr453, an aromatic residue essential for binding cadaverine, the feedback inhibitor of CadC activation [33]. It was possible that all three mutants were incapable of binding cadaverine, which would result in elevated expression of *cadAB*, thus increasing cadaverine production and acid tolerance. Some effect on the CadC response to cadaverine was detected, although the effects were small. Transcriptional analysis of *cadA* expression at pH 5 and pH 7 revealed that all three CadC mutants are constitutively active under both conditions, whereas WT CadC only activates *cadAB* expression at pH 5. Mutations of several CadC residues have previously been identified that activate *cadBA* expression irrespective of external pH [33, 62]. The cluster of CadC mutations identified in this study is distinct from these and thus highlights additional residues crucial for the conformational switch to an activated CadC. *E. coli* Δ*cadA* or Δ*cadC* mutants proved hypersensitive to EDTA under acidic conditions (pH 5–6). Introduction of the WT *cadC* on plasmids to a Δ*cadC* strain restored tolerance of EDTA at pH 5 and this was further enhanced when plasmids carrying the *cadC* mutants were introduced. Significantly, all three of the CadC mutants were more effective than the WT, either plasmid-encoded or the original mutants, at keeping the pH of the growth media close to neutral. Maintenance of a more physiological environment might bolster EDTA tolerance, although *E. coli* WT susceptibility to this chelant is not significantly different across the pH 5–7 range (Fig. 8A; Fig. S17a). The improvements proffered by the CadC mutants over the WT expressed from plasmids may be more evident due to incomplete restoration of CadC levels compensating for the absence of *cadC* in these strains. In any case, a *cadC* mutation was selected in the absence of chelating agents and this control strain, JN186, failed to show improved resistance to EDTA and DTPMP (Fig. S19).

Why EDTA should have enhanced toxicity against *E. coli* at pH 4 is not clear, although the acidic conditions could potentially exacerbate damaging effects of this chelator on the bacterial outer membrane [17, 18, 63–67]. The discovery that CadC missense mutations also arose during experimental evolution with two antibacterials with known membrane-damaging effects, triclosan [50] and carvacrol [51], was potentially significant. However, these mutations differed widely in position and only the carvacrol-isolated mutant occupied the CadC dimer interface. None of the *cadC* mutants isolated in this study displayed resistance to either carvacrol or triclosan under acidic or neutral conditions (Fig. S20). Since a CadC M448V mutation was isolated without exposure to EDTA or DTPMP, there must be an alternative explanation for the appearance of these mutants. Significantly, all three experimental evolution studies with chelators, carvacrol and triclosan utilized LB broth. This culture medium lacks buffering capacity and is therefore subject to changes in pH as a result of bacterial growth, which tends to acidify its surroundings. Hence these *cadC* mutations most likely promote *E. coli* growth and survival during long-term cultivation, though how this is achieved presumably differs depending on the precise location and nature of the amino acid substitutions.

Finally, the observed resistance mechanisms fit with the known effect of the two chelants on cellular metal levels [16]. Since growth in the presence of EDTA can be restored by improving zinc distribution, this metal would seem to be primarily responsible for growth inhibition by EDTA. However, it cannot be fully disentangled from coinciding reductions in iron and manganese. These complexities should be taken into consideration when using EDTA and other chelants as ‘specific’ metal binders. Improvements in iron uptake mechanisms would fit with DTPMP functioning as a chelator that principally affects this metal, at least at low concentrations. One can envisage the two differing tolerance mechanisms arising when bacteria are experiencing growth restriction, either by nutritional immunity or by exposure to metal-sequestering antimicrobials. The potential for bacterial resistance needs to be considered for applications of metal chelators in consumer goods or in a therapeutic context, especially against typically intransigent Gram-negatives. However, the results provided here are encouraging and suggest that disrupting the finely tuned balance of metal homeostatic systems also has detrimental consequences for *E. coli* fitness. Should evolvable resistance prove problematic, it could be overcome either by utilizing chelator pairs with contrasting metal selectivities [16] or in combinations with other antimicrobial agents [21, 68–71].

## Supplementary Data

Supplementary material 1
