# Supplementary material for: A critical role for iron and zinc homeostatic systems in the evolutionary adaptation of Escherichia coli to metal restriction
Source: Microb Genom. 2023 Dec 6;9(12):001153. doi: 10.1099/mgen.0.001153 (PMC10763504; doi:10.1099/mgen.0.001153)
Supplement: Supplementary material 1 [file mgen-9-1153-s001.pdf]

Supplementary Material

A critical role for iron and zinc homeostatic systems in the evolutionary adaptation of *Escherichia coli* to metal restriction

Joy R. Paterson, Joshua M. Wadsworth, Ping Hu and Gary J. Sharples

Table S1. Chelating agents used in this study.

Synonym	Formal Name (s)	CAS Number	Source	Solvent
CAT	Catechol, 1,2-dihydroxybenzene	120-80-9	Sigma-Aldrich	Water
CHA	Caprylhydroxamic acid, octanohydroxamic acid	7377-03-9	Fluorochem	DMSO
CHL	Chloroxine, dichlorchinolinolum,	773-76-2	Sigma-Aldrich	DMSO
DTPA	Diethylenetriaminepentaacetic acid	67-43-6	Sigma-Aldrich	Water
DTPMP	Diethylenetriaminepentakis(methylphos phonic acid)	15827-60-8	Sigma-Aldrich	N/A
EDTA	Ethylenediaminetetraacetic acid	60-00-4	Melford	Water
FA	Fusaric acid, 5-butylpyridine-2-carboxylic acid, fusarinic acid	536-69-6	Sigma-Aldrich	DMSO
GLDA	N,N-bis(carboxymethyl)-L-glutamic acid (tetrasodium salt)	51981-21-6	Biosynth Carbosynth	Water
HBED	N,N'-di(2-hydroxybenzyl)ethylene diamine-N,N'-diacetic acid (monohydrochloride hydrate)	35369-53-0	Strem Chemicals	DMSO
HNK	2-Hydroxy-6-(propan-2-yl)cyclohepta-2,4,6-trien-1-one, β-Thujaplicin, hinokitiol	499-44-5	Sigma-Aldrich	DMSO
HPNO	2-hydroxypyridine-N-oxide	13161-30-3	Biosynth Carbosynth	Water
MGDA	Methylglycinediacetic acid, N-(1-carboxylatoethyl)iminodiacetate (trisodium hydrate)	164462-16-2	TCI	Water
PO	Piroctone olamine, 1-hydroxy-4-methyl-6-(2,4,4-trimethylpentyl)-2(1H)-pyridone ethanolammonium salt	68890-66-4	Combi-Blocks	DMSO
TPEN	N,N,N',N'-tetrakis(2-pyridinylmethyl)-1,2-ethanediamine	16858-02-9	Cayman Chemical	Ethanol
TRO	Tropolone, 2-Hydroxy-2,4,6-cycloheptatrien-1-one	533-75-5	Sigma-Aldrich	DMSO

DTPA and EDTA were solubilised by addition of NaOH to pH 8. For chelants provided in liquid form, no solvent was required prior to testing.

Table S2. Oligonucleotides used for qPCR in this study.

Primer name	Nucleotide sequence (5'-3')	Length	%G+C	Product (bp)
*yeiR*_F	AAAACTTCCGTGACCAGCTG	20	50	110
yeiR _R	ACCATTTTGCTGCCACCAAC	20	50	
*fepA*_F	AAACGACCTTCACCTGGTAC	20	50	110
*fepA*_R	GCCAACAATGCTGTAAGGAC	20	50	
*cadA*_F	CCTGATGATGATGAGCGATG	20	50	111
*cadA*_R	TTAGCAATGGTAGCGTGCTG	20	50	
*mntS*_F	GTATGCGCGTGTTTAGTCATTC	22	45	105
*mntS*_R	TATCGGAAGGTTTATCTTGCTG	22	45	
*fepD*_F	TGCAAACCCTCACCCGAAAC	20	55	111
*fepD*_R	GCGCGGAAGAGTAACCAAACAG	22	55	
*znuA*_F	GTTTGGACTGACACCGCTTG	20	55	111
*znuA*_R	ACGCAGGTTGCTTTTTGCTC	20	50	
*zntA*_F	CGAAGCACAGGTTGCTGAAC	20	50	114
*zntA*_R	CCGGCAGCGCAAATCAATAC	20	55	
*rpoD*_F	GTGGCTTGCAGTTCCTTGAC	20	55	108
*rpoD*_R	AGGTTGCGTAGGTGGAGAAC	20	55	

Primers for amplification of cDNA from *mntS*, *fepD*, *znuA*, *zntA* and *rpoD* are identical to those used previously for work on *E. coli* JM109 [1] and match relevant gene sequences from BW25113.

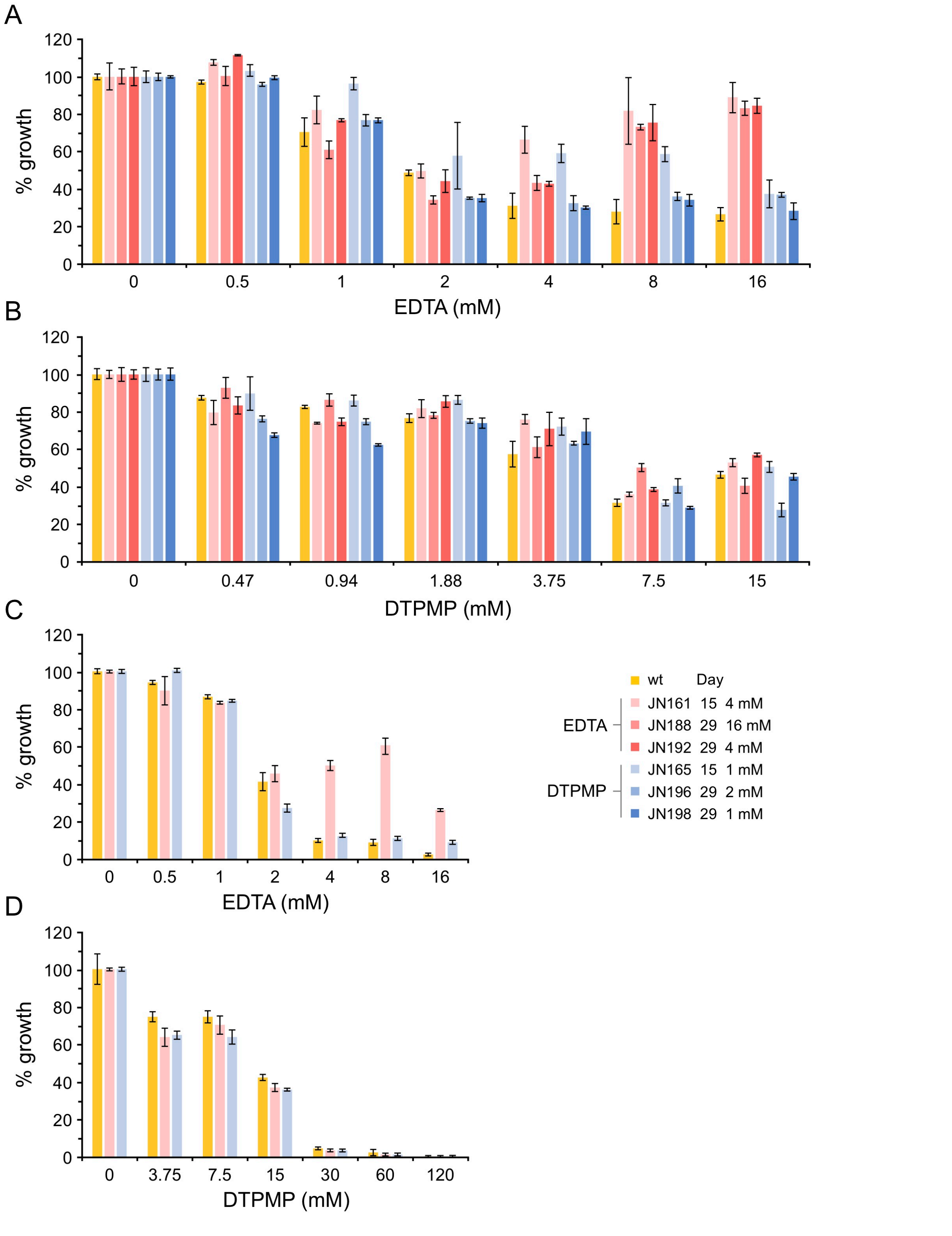

Fig. S1. Susceptibility of wild-type and chelant-treated strains to EDTA (A and C) or DTPMP (B and D). Two-fold serial dilutions of each chelant were mixed with each strain and incubated at 37°C with shaking at 150 rpm for 16 h. Growth was measured at $OD_{600\ nm}$ at the endpoint and normalised against controls without chelant to give the percentage growth. The two Day 15 isolates were checked for chelant resistance at the mid-way point of the experiment (C and D). Results are independent replicates of the data shown in Figure 1B and C and are the mean and standard deviation of experiments performed in triplicate.

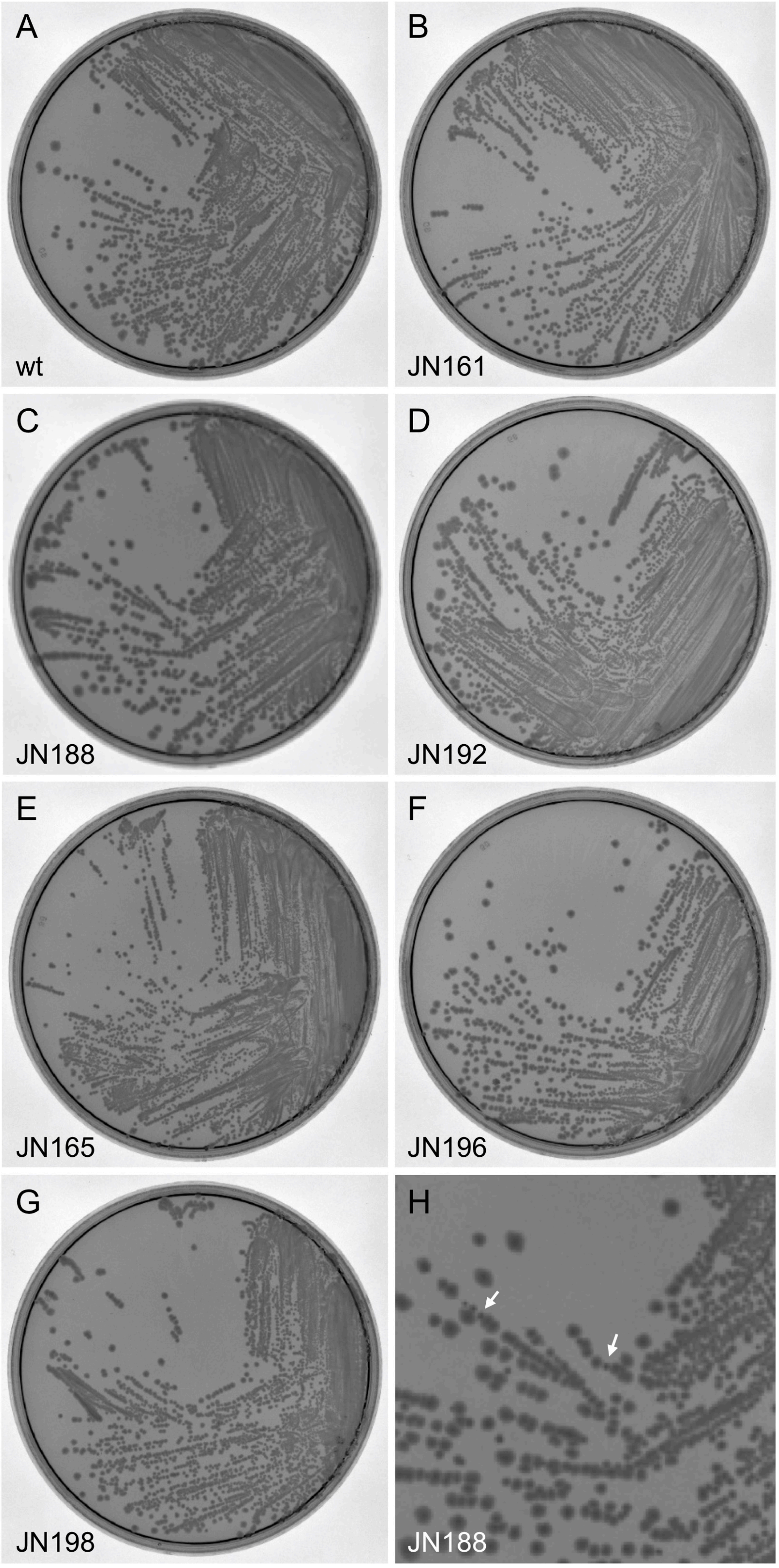

Fig. S2. Colony morphology of *E. coli* chelant-selected mutants. Isolated strains were streaked and grown on LB agar at 30°C for 16 h prior to imaging. BW25113 wild-type (A), JN161 (B), JN188 (C), JN192 (D), JN165 (E), JN196 (F) and JN198 (G). A close-up of JN188 colony morphology is included to highlight the large and small colony variants (H). Large and small colonies from these strains streaked onto fresh LB agar plates produced both size variants.

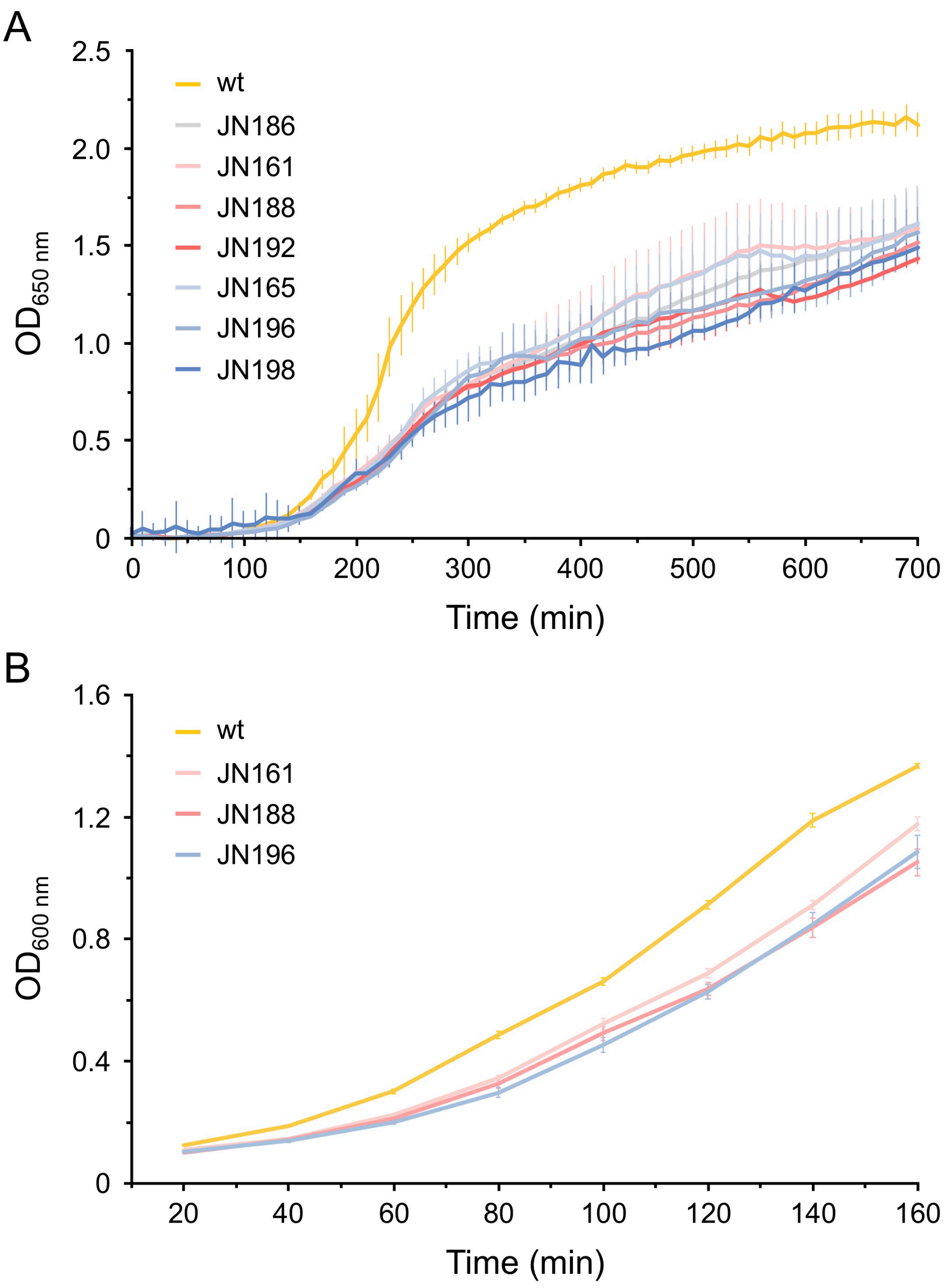

Fig. S3. Growth curves of *E. coli* chelant-selected mutants. (A) Strains were cultivated in 100 µl volumes in LB broth at 37°C and absorbance monitored at $OD_{650\ nm}$. The results are the mean and standard deviation of a single experiment performed in triplicate. (B) Selected strains were also grown in 50 ml volumes LB broth at 37°C and absorbance monitored at $OD_{600\ nm}$. Those isolated in the presence of EDTA (JN161, JN188 and JN192) are highlighted in shades of red, while those isolated in the presence of DTPMP (JN165, JN196, JN198) are in blue. A control selected in the absence of chelant (JN186) is shown in grey (A). The results are the mean and standard deviation of four independent repeats.

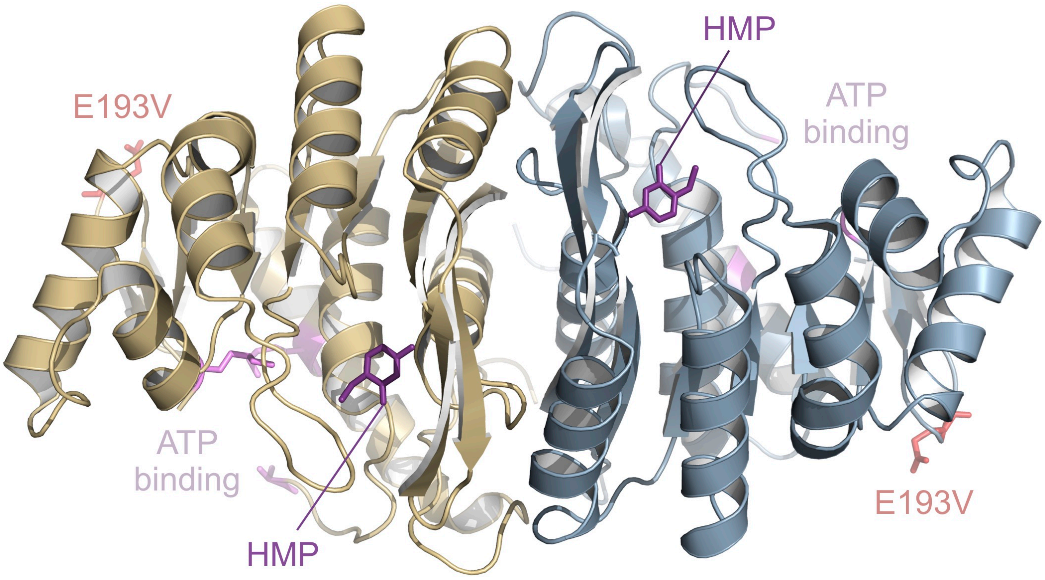

Fig. S4. Location of the E193K mutation in ThiD.Structure of *Salmonella enteriditis* serovar Typhimurium ThiD protein in complex with hydroxymethylpyrimidine (HMP; Protein Data Bank (PDB): 1JXI; [2]. Residues (Lys176, Arg202 and Lys237) involved in ATP binding are shown in pink. The location of the Glu193Val substitution found in the EDTA-resistant strains JN161 and JN192 is highlighted in red. This residue is conserved and lies on the surface of hydroxymethylpyrimidine (HMP) kinase [2], an enzyme involved in thiamine biosynthesis; this residue is not located near the HMP binding site or dimer interface but its mutation could potentially affect the arrangement of residues required for ATP binding [2, 3]. The protein structure was generated in Pymol.None of the additional mutations, listed in Figure 2, are present in every chelant-selected strain and are therefore unlikely to make major contributions to either EDTA or DTPMP tolerance. However, these changes are discussed below for completeness. AlsR (RpiR) is a transcriptional repressor of the D-allose uptake and catabolism regulon. Although the AlsR Phe138Val mutation found in JN165 occurs in the SIS sugar isomerase domain [4], this portion of the domain is not especially well conserved. JN196 is the only strain to carry a Glu317Lys substitution in YahJ, an uncharacterised metal-dependent amidohydrolase that appears to possess a signal sequence for export. The amino acid substitutions in RhsA (Thr1042Ser) and YdbA (Asn493Lys and Ile485Met) are found in JN196 and JN192, respectively, alongside multiple synonymous changes that are likely due to frequent genetic rearrangements between the repeated sequences characteristic of these two proteins. The *stfE* gene, encoding an uncharacterised phage tail fibre protein, carries multiple frameshift mutations in four out of six chelant-selected strains that are likely to inactivate the gene. However, it is unlikely that loss of StfE function nor any of the other substitutions listed above are primary factors in chelant tolerance, although they may help in combination with the other changes present in each mutant. Upstream mutations could potentially amplify gene expression levels to help alleviate chelant toxicity. The mutations adjacent to *glyV*, *flxA* (downstream of the convergently transcribed *flxA* and *ydfW* phage genes), *ldrA* (also in a downstream region) and *lacZ* do not appear to affect any key transcriptional elements and are therefore unlikely to contribute to chelant tolerance. BW25113 has an insertion (Δ*lacZ4787*) that disrupts the *lacZ* gene [5].

The genetic changes upstream of *fimA* in JN165 (Fig. 2) result from site-specific flipping of the 314 bp invertible segment responsible for type I fimbrial gene phase variation [6, 7]. In JN165, this inverts the orientation of the promoter to switch on the expression of the entire *fim* operon (*fimAICDFGH*). Although JN165 was not one of the EDTA-resistant strains associated with colony size variation, it does highlight that genetic changes affecting the *fim* operon are the likely source of the colony size variants in these strains (Fig. S2).

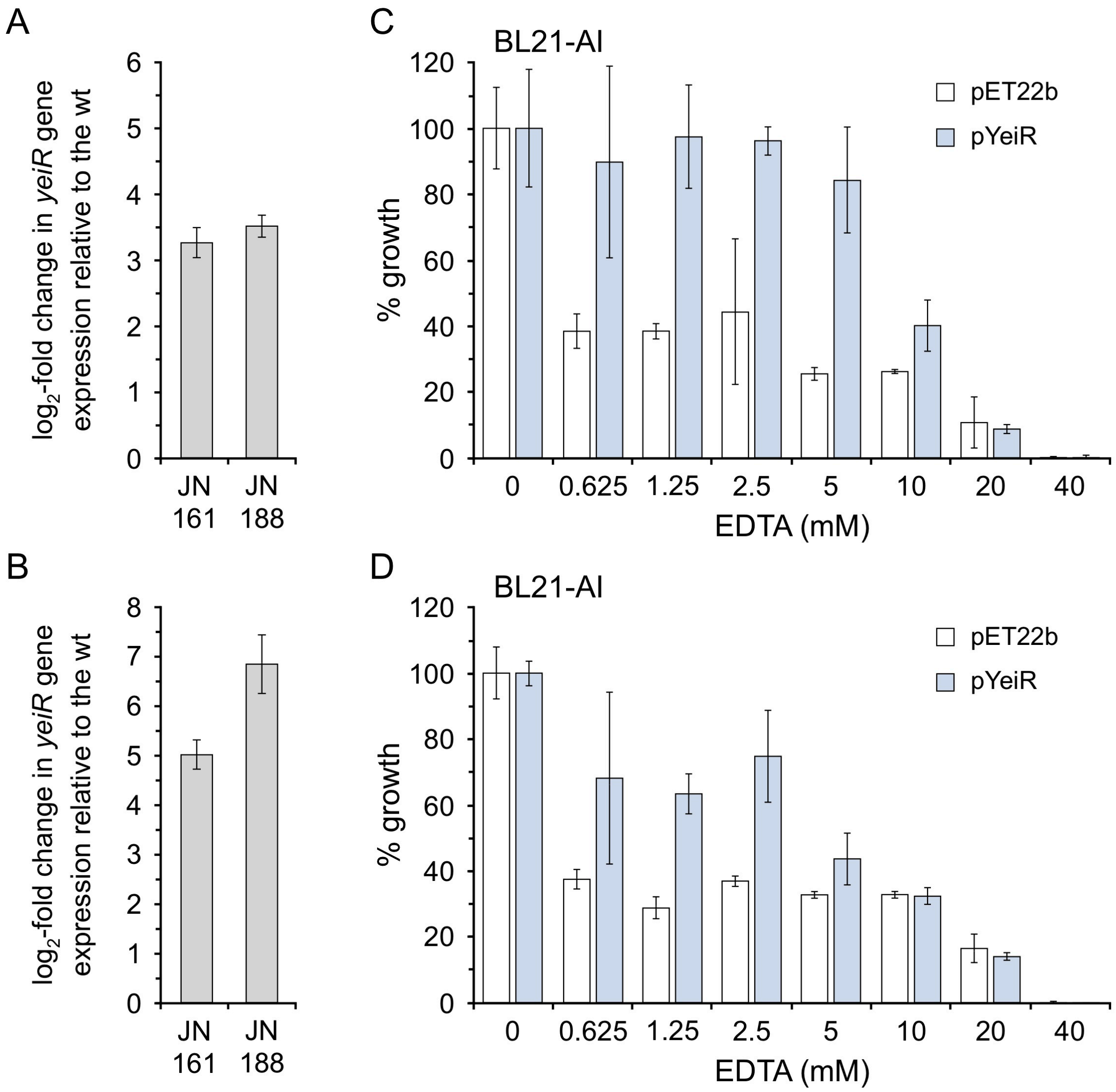

Fig. S5. Elevated expression of the YeiR zinc-dependent GTPase is responsible for improved growth in the presence of EDTA. (A and B) Mutations in EDTA-selected strains upregulate *yeiR* gene expression. qPCR was used to monitor expression levels of the *yeiR* gene in JN161 and JN188 strains relative to the wt; *rpoD* was employed as a reference gene in all samples. Results are independent replicates of the data shown in Figure 3C and are the mean and standard deviation of experiments performed in triplicate. (C and D) Overexpression of YeiR in *E. coli* promotes resistance to EDTA. BL21-AI cells carrying pYeiR, the *yeiR* gene inserted into pET22b, were grown in LB at 37°C and expression was induced by the addition of 1 mM IPTG and 0.2% arabinose and incubated for a further 16 h. BL21-AI carrying the pET22b vector control was run in parallel. Growth was measured at $OD_{600\ nm}$ at the endpoint and normalised against controls without chelant to give the percentage growth. Results are independent replicates of the data shown in Figure 3D and are the mean and standard deviation of experiments performed in triplicate.

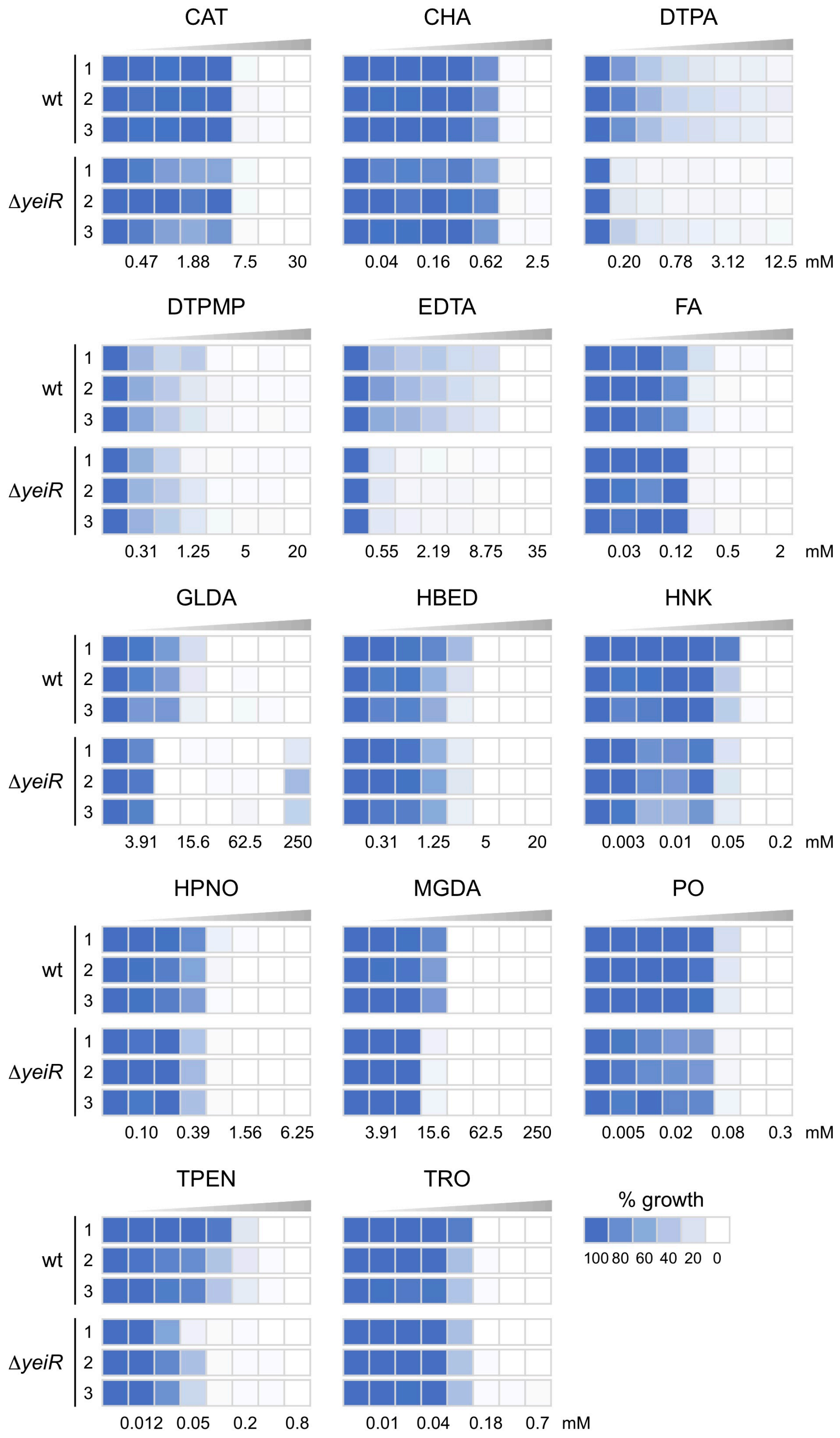

Fig. S6. Susceptibility of *E. coli* wt and a *yeiR* mutant to different metal chelating agents. Diluted cultures of *E. coli* wt (BW25113) and the isogenic Δ*yeiR* mutant were mixed with two-fold serial dilutions of each chelant and incubated at 37°C with shaking at 150 rpm for 16 h. The extent of growth was measured at $OD_{600\ nm}$ at the end point and normalised against controls without chelant to give the percentage growth. Three independent replicates (labelled 1-3) are shown for each strain, incorporating one of the data sets shown in Figure 4 and Figure S6, and are the mean of experiments performed in triplicate with growth indicated by conditional formatting (values capped at 100% growth). At the highest concentration GLDA caused cell aggregation in the *yeiR* strain but not the wt.

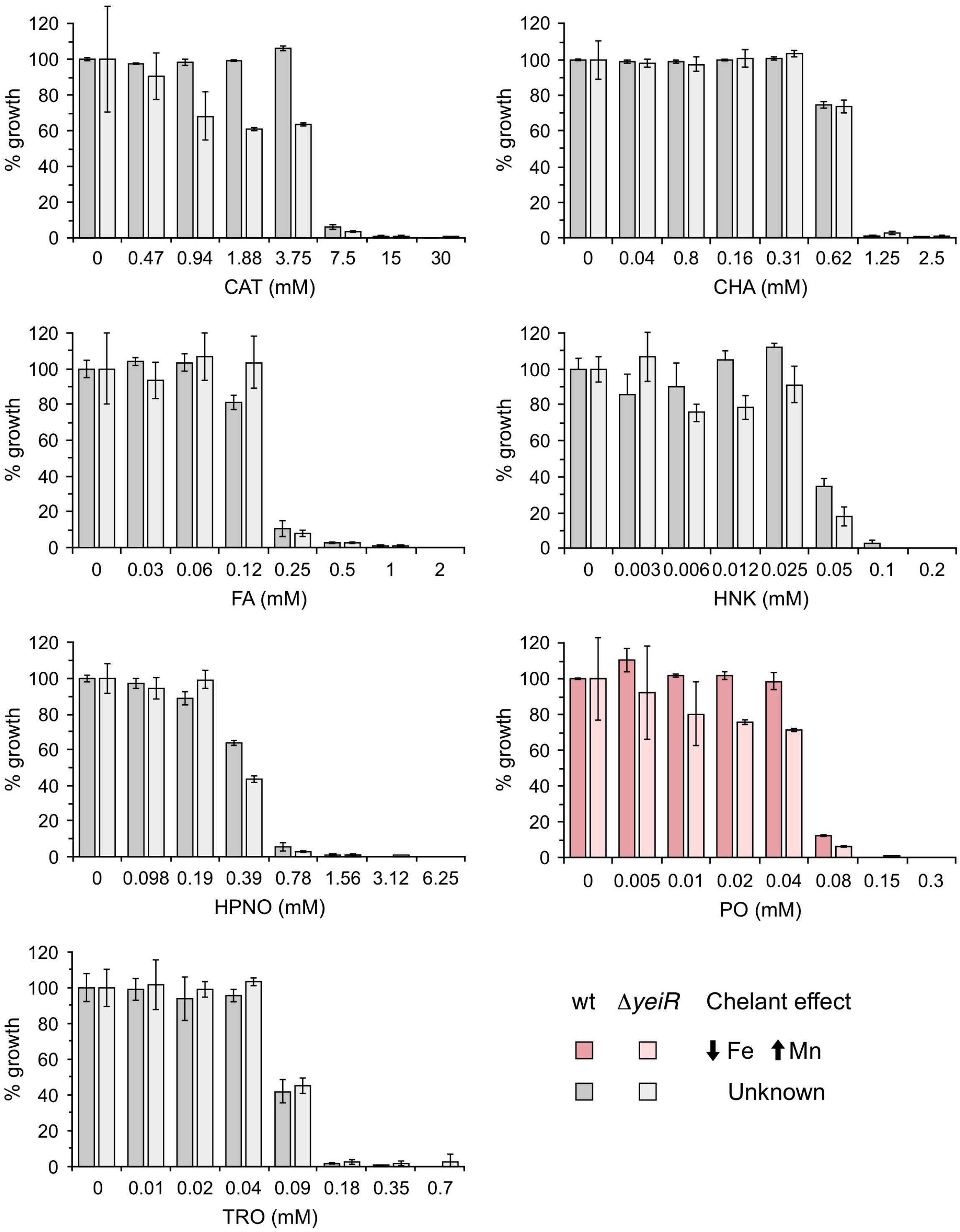

Fig. S7. Susceptibility of *E. coli* wt and a *yeiR* mutant to different metal chelating agents. Diluted cultures of *E. coli* wt (BW25113) and the isogenic Δ*yeiR* mutant were mixed with two-fold serial dilutions of each chelant and incubated at 37°C with shaking at 150 rpm for 16 h. The extent of growth was measured at $OD_{600\ nm}$ at the end point and normalised against controls without chelant to give the percentage growth. Results represent the mean and standard deviation of an experiment performed in triplicate. Two additional independent repeats yielded similar results (Fig. S5). PO effects on *E. coli* cellular metal content are indicated based on previous ICP-MS analysis [8]; CAT and CHA showed no change in metal content in these studies and the other four chelants have yet to be tested.

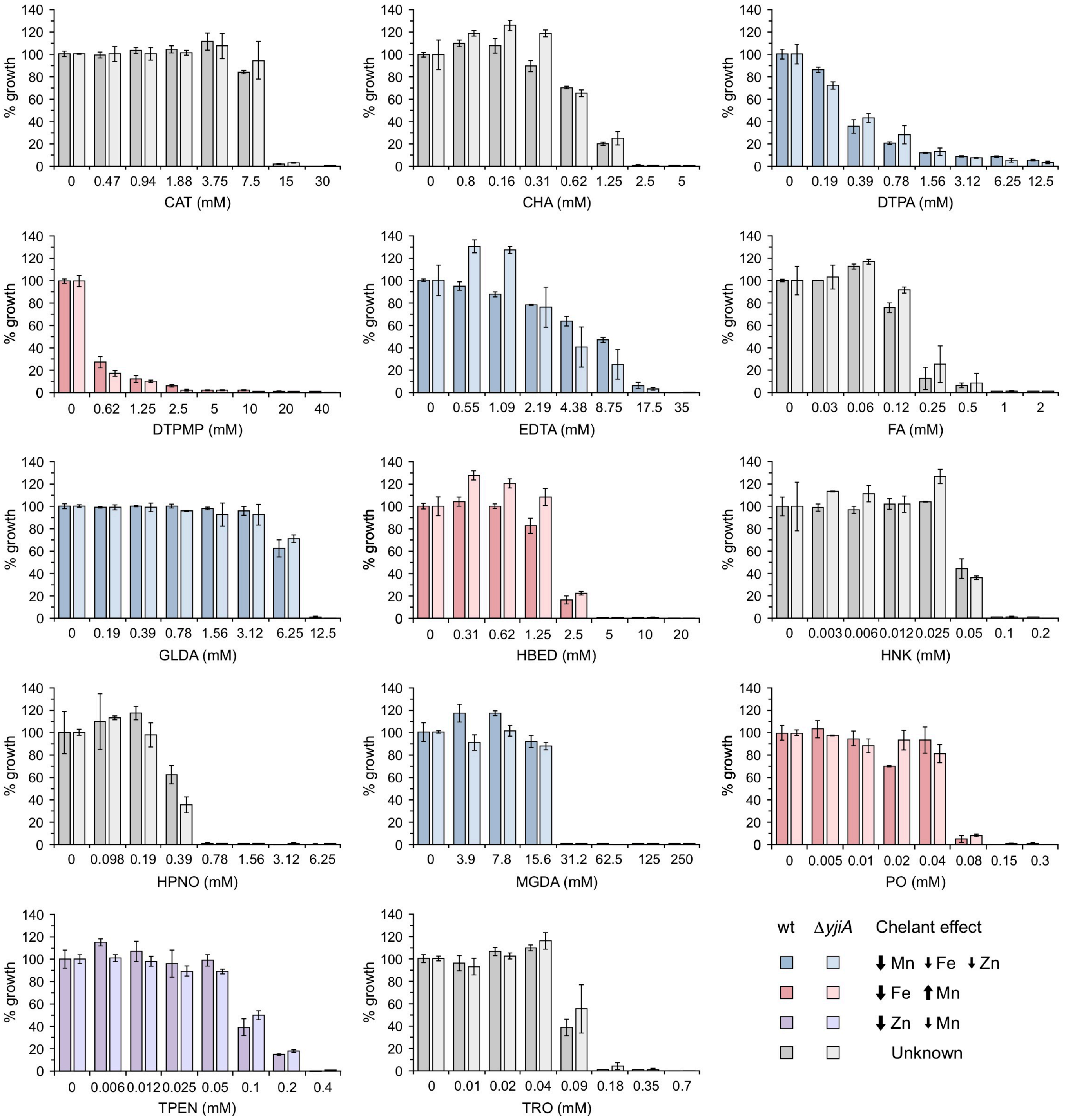

Fig. S8. Susceptibility of *E. coli* wt and *yjiA* mutant to different metal chelating agents. Diluted cultures of *E. coli* wt (BW25113) and the isogenic Δ*yjiA* mutant were mixed with two-fold serial dilutions of each chelant and incubated at 37°C with shaking at 150 rpm for 16 h. The extent of growth was measured at $OD_{600\ nm}$ at the end point and normalised against controls without chelant to give the percentage growth. Results represent the mean and standard deviation of an experiment performed in triplicate. Two additional independent repeats yielded similar results (Fig. S8). Chelant effects on *E. coli* cellular metal content are indicated based on previous ICP-MS analysis [8].

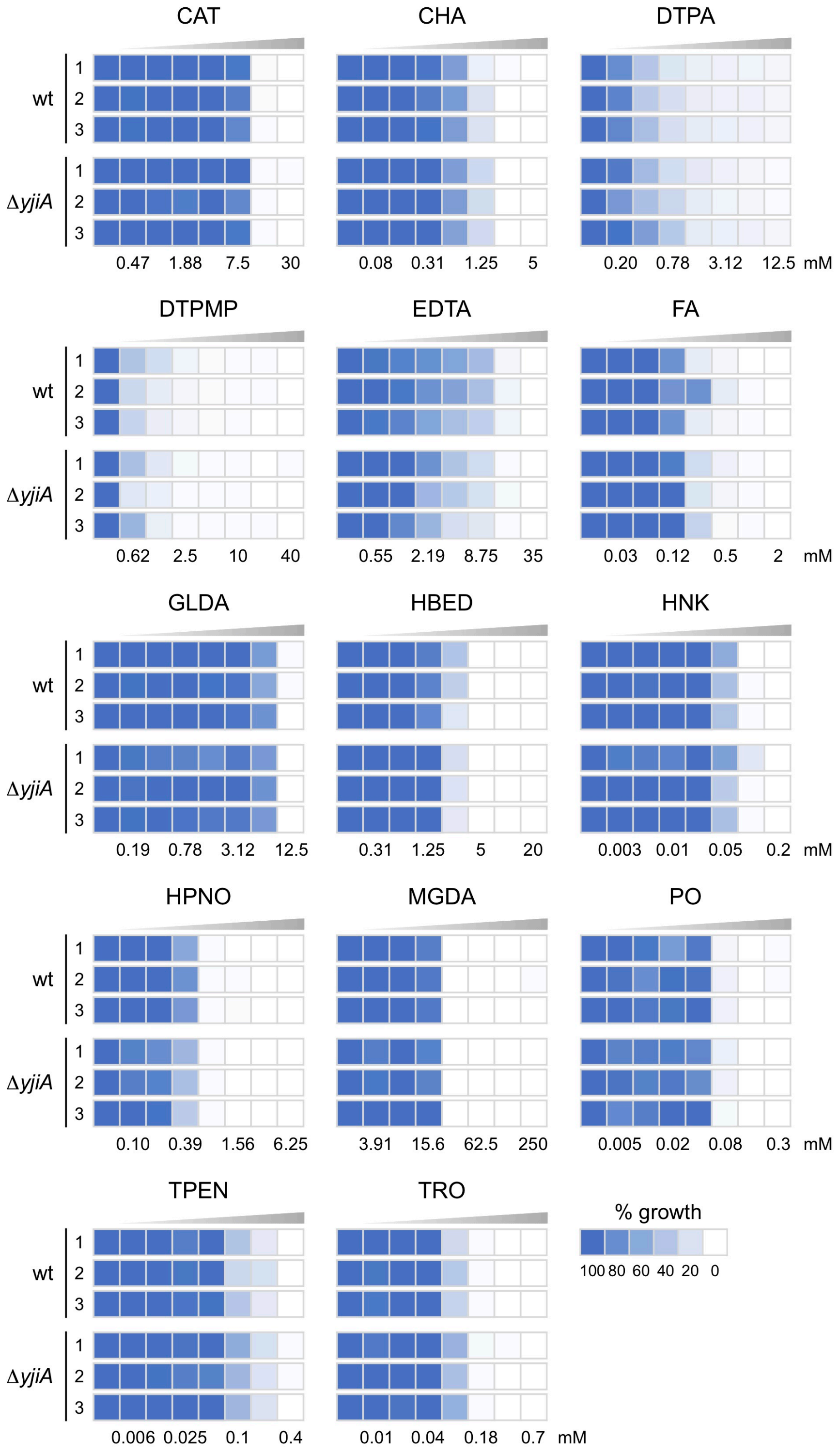

Fig. S9. Susceptibility of *E. coli* wt and *yjiA* mutant to different metal chelating agents. Diluted cultures of *E. coli* wt (BW25113) and the isogenic Δ*yjiA* mutant were mixed with two-fold serial dilutions of each chelant and incubated at 37°C with shaking at 150 rpm for 16 h. The extent of growth was measured at $OD_{600\ nm}$ at the end point and normalised against controls without chelant to give the percentage growth. The three independent replicates (labelled 1-3) are shown, incorporating one of the data sets shown in Figure S7, and are the mean of experiments performed in triplicate with growth indicated by conditional formatting (values capped at 100% growth).

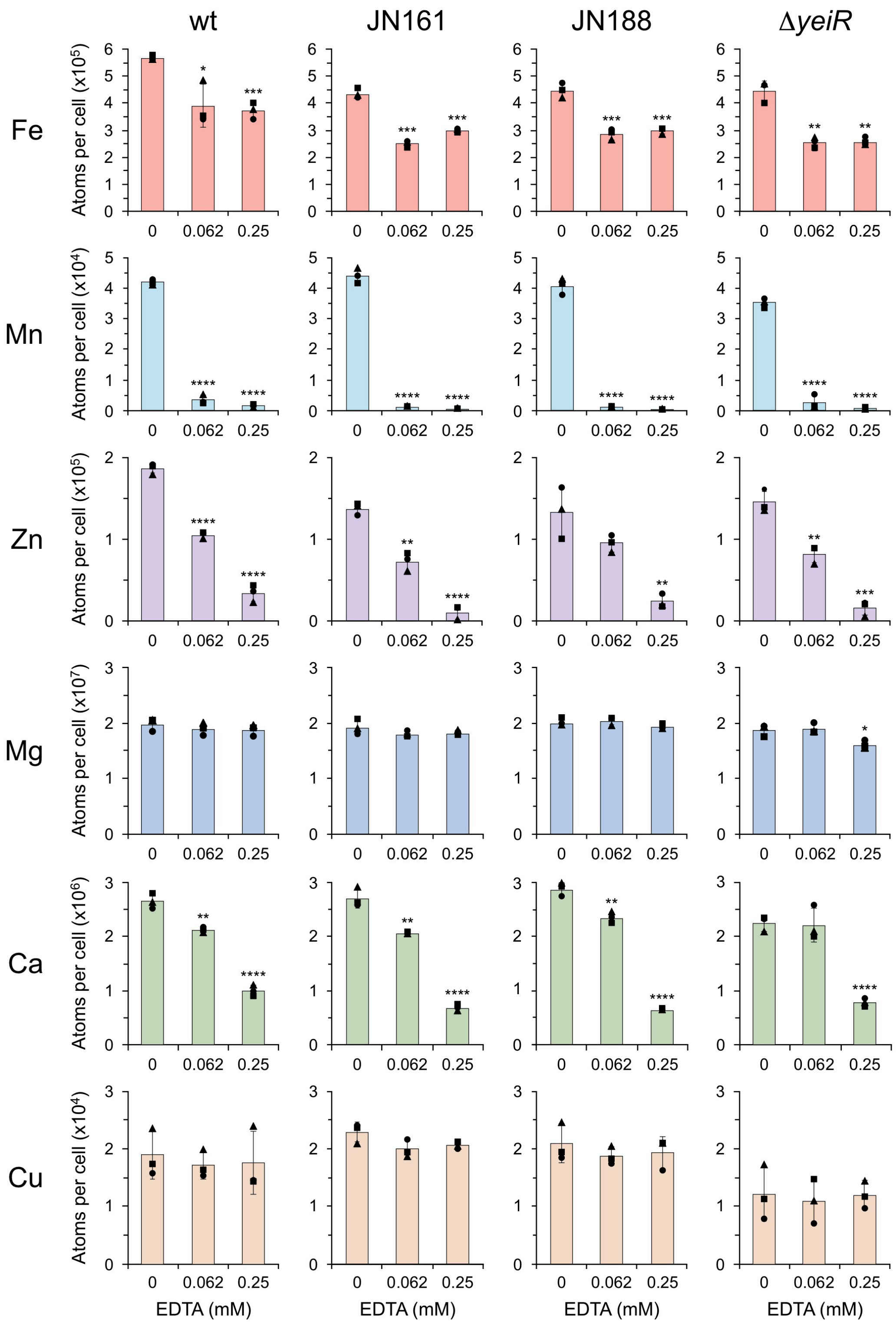

Fig. S10. Effect of EDTA on cellular metal composition of *E. coli* wt, JN161, JN188 and Δ*yeiR* strains. Bacteria were grown in 50 ml of LB pH7 to early log-phase in a shaking incubator (125 rpm) at 37°C. EDTA was added at the outset to produce a growth inhibition of 10-25% and untreated controls set up in parallel. Amounts of each metal were determined by ICP-MS and presented in number of atoms per cell. Data are the mean and standard deviation of 3 independent experiments. Different symbols are used for each data

set. The t-test was used to compare each chelant concentration against the untreated control, *$P < 0.05$, **$P < 0.01$, ***$P < 0.001$ and ****$P < 0.0001$ (n = 3).

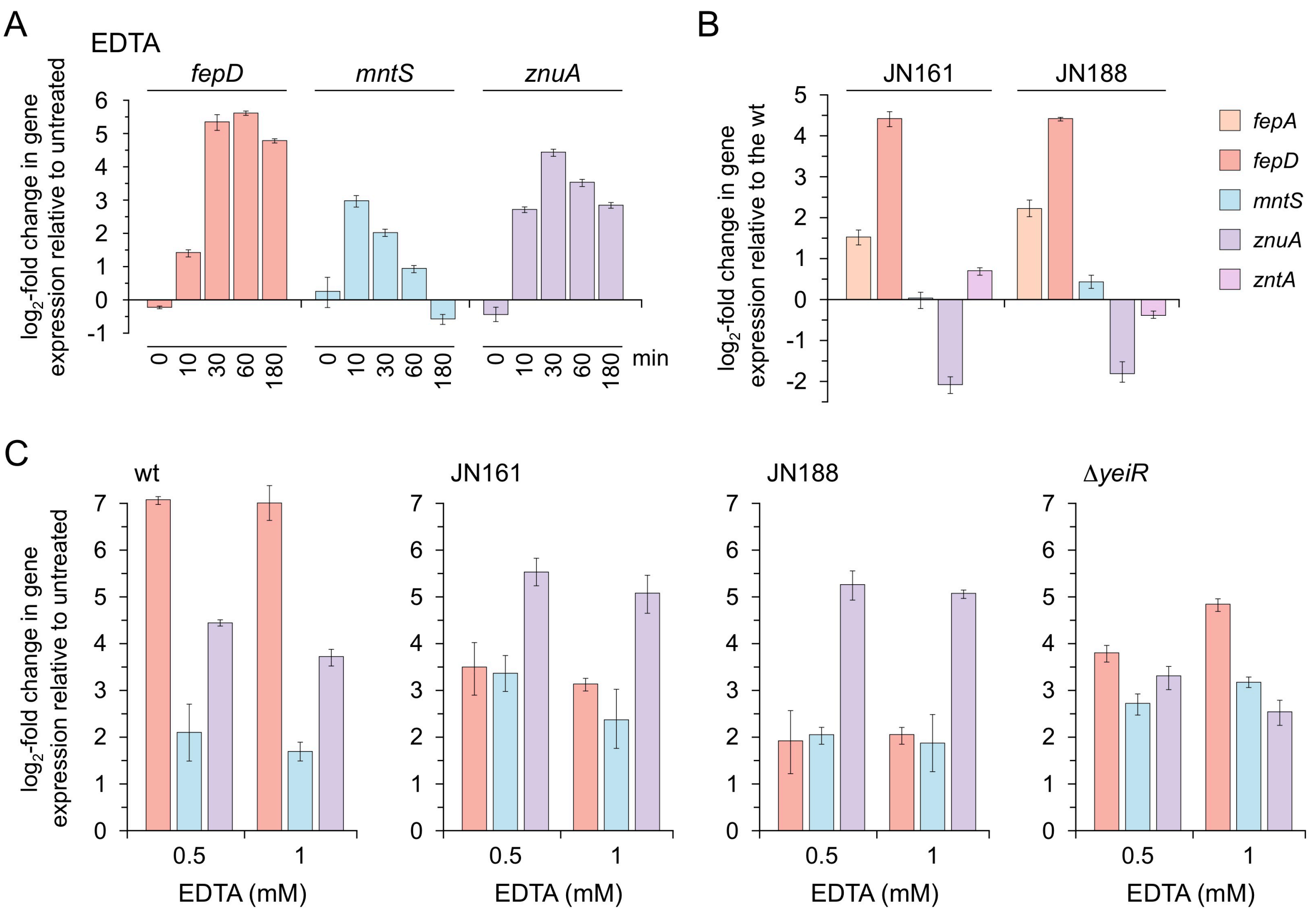

Fig. S11. Effect of *yeiR* promoter mutations in EDTA-selected strains on expression of metal-regulated sensors. (A) Relative metal sensor gene expression in the BW25113 wt in the presence of EDTA at different time intervals. qPCR was used to monitor expression levels of *fepD*, *mntS*, *znuA* and *rpoD* as a reference gene. Bacteria were grown to an $OD_{600\ nm}$ of 0.3 and 1 mM EDTA added at samples removed for analysis at the indicated intervals. (B) Relative metal sensor gene expression between mutants and wt in the absence of chelant. qPCR was used to monitor expression levels of each metal-regulated gene in strains JN161 and JN188 relative to the wt. (C) Relative metal sensor gene expression between mutants and wt in the presence of EDTA. qPCR was used to monitor expression levels of *fepD*, *mntS* and *znuA* in the BW25113 wt, JN161, JN188 and JW2161 (Δ*yeiR*::*kan*) exposed to either 0.5 or 1 mM EDTA relative to untreated controls. *rpoD* was employed as a reference gene in all samples. Data in B and C are the mean and standard deviation of an experiment performed in triplicate, independent repeats of those shown in Figure 5B and C. Genes targeted by qPCR are colour coded as indicated in the key depicted in B.

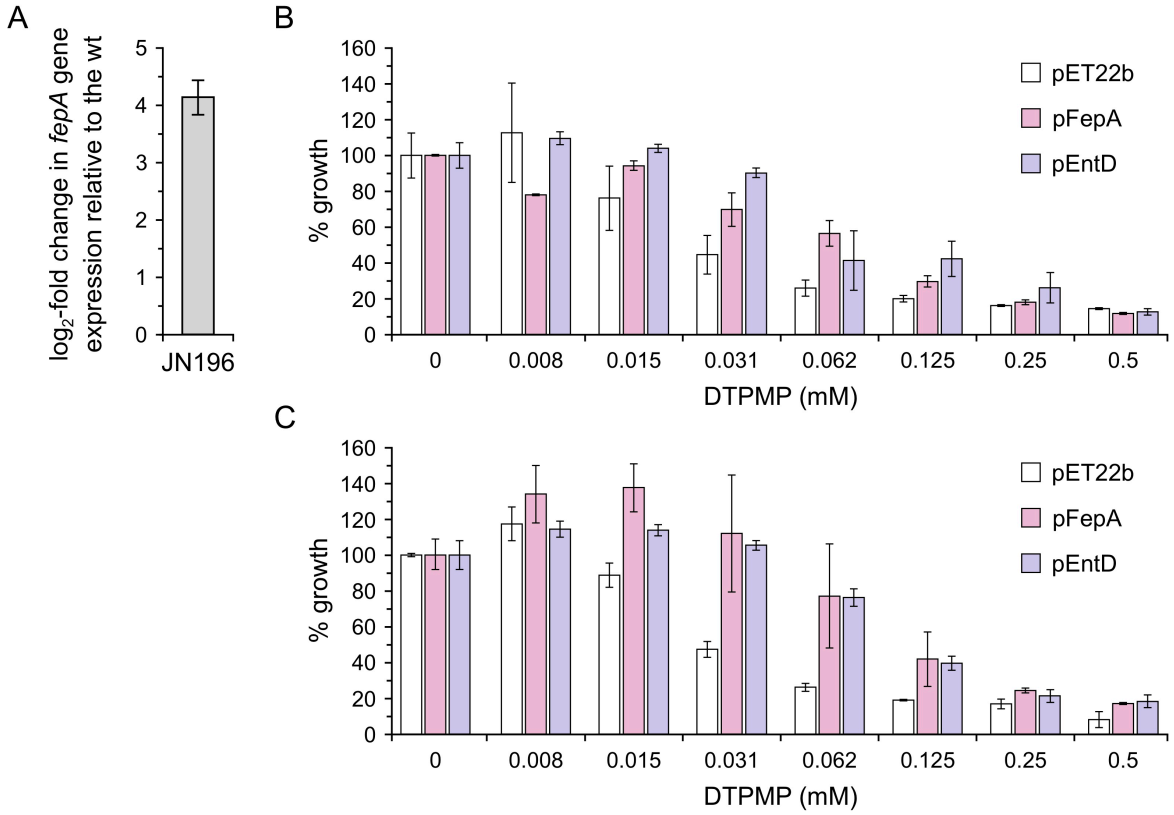

Fig. S12. Elevated expression of *fepA* or *entB* improve growth in the presence of DTPMP. (A) The promoter mutation in a DTPMP-selected strain upregulates *fepA* gene expression. qPCR was used to monitor expression levels of the *fepA* gene in JN196 strains relative to wt; *rpoD* was employed as a reference gene. The data is an independent replicate of that shown in Figure 6C and is the mean and standard deviation of an experiment performed in triplicate. (B and C) Overexpression of FepA or EntD proteins in *E. coli.* BL21-AI cells carrying pFepA or pEntD, the *fepA* or *entD* genes, respectively, inserted into pET22b, were grown in LB at 37°C and expression induced by addition of 0.5 mM IPTG and 0.1% arabinose and incubated for a further 16 h. BL21-AI carrying the pET22b vector control was run in parallel. Growth was measured at $OD_{600\ nm}$ at the endpoint and normalised against controls without chelant to give the percentage growth. Results are independent replicates of the data shown in Figure 6D and are the mean and standard deviation of experiments performed in triplicate.

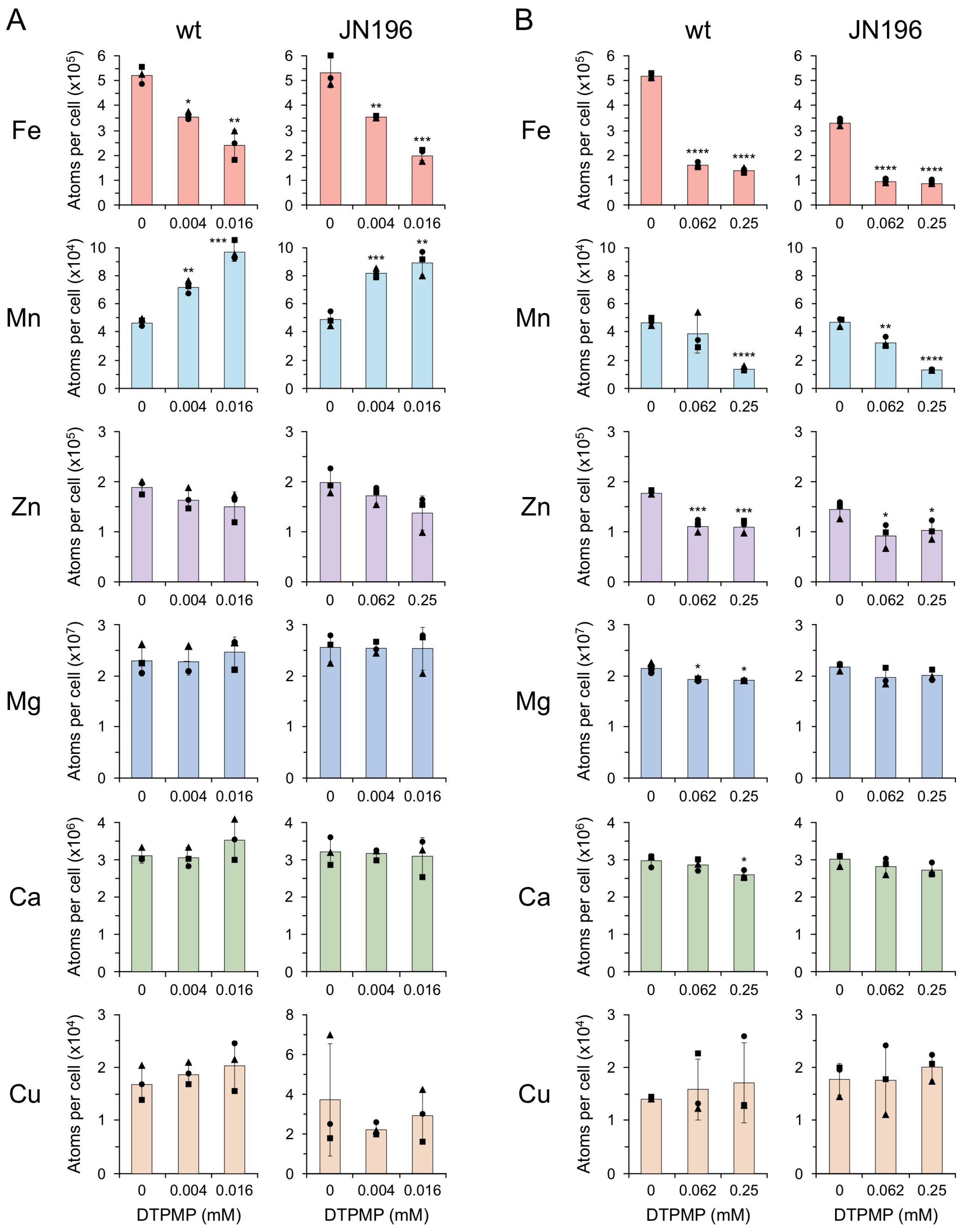

Fig. S13. Effect of DTPMP on cellular metal composition of *E. coli* wt and JN196. Bacteria were grown in 50 ml of LB pH7 to early log-phase in a shaking incubator (125 rpm) at 37°C. DTPMP was added at the outset at low (A) and high (B) concentrations to produce a growth inhibition of 10-25% and untreated controls set up in parallel. Amounts of each metal were determined by ICP-MS and presented in number of atoms per cell. Data are the mean and standard deviation of 3 independent experiments. Different symbols are used for each data set. The t-test was used to compare each chelant concentration against

the untreated control, *$P < 0.05$, **$P < 0.01$, ***$P < 0.001$ and ****$P < 0.0001$ (n = 3). One of the data points in the control for Cu with JN196 was much higher than the others and the scale has been adjusted to reflect this outlier.

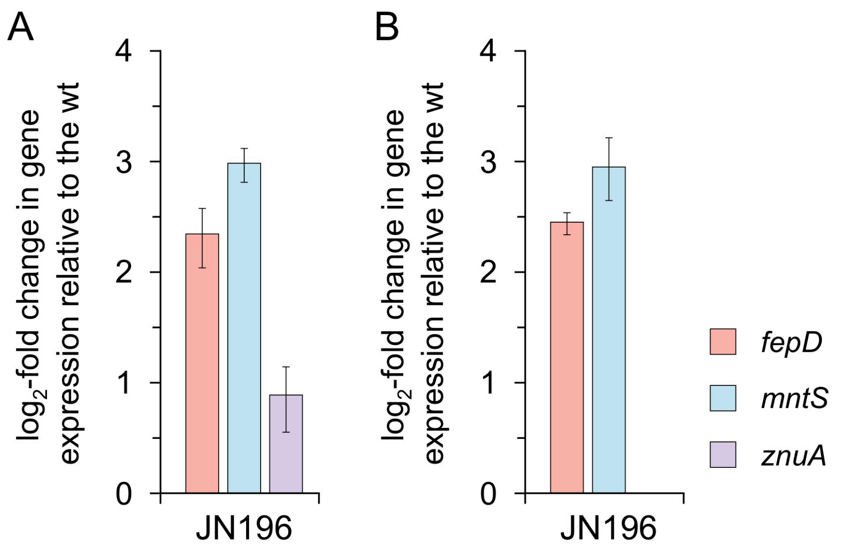

Fig. S14. Relative metal sensor gene expression between wt and representative DTPMP-selected strain in the absence of chelant. qPCR was used to monitor expression levels of *fepD*, *mntS*, *znuA* and *rpoD* as a reference gene in BW25113 wt and JN196. Data are the mean and standard deviation of an experiment performed in triplicate (A). An independent repeat experiment showed similar results (B). Genes targeted by qPCR are colour coded as indicated in the key shown on the right.

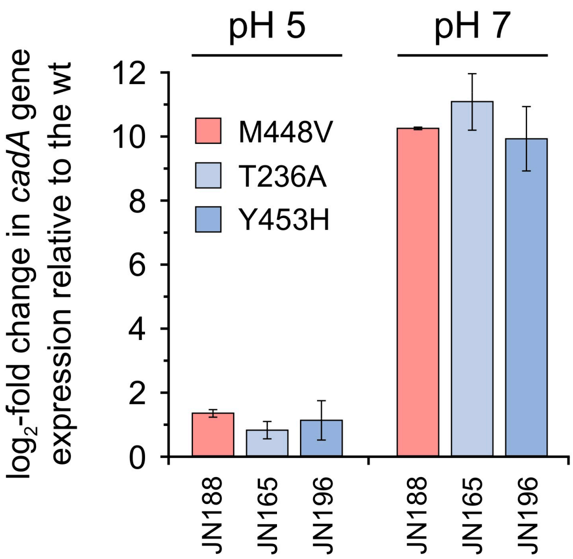

Fig. S15. Mutations in the CadC protein result in constitutive expression of the *cadAB* operon. Expression of *cadA* in chelant-selected strains carrying mutations in *cadC*. qPCR was used to monitor expression levels of the *cadA* gene in JN165, JN188 and JN196 relative to the wt; *rpoD* was employed as a reference gene. Results are an independent replicate of the data shown in Figure 7E and are the mean and standard deviation of experiments performed in triplicate.

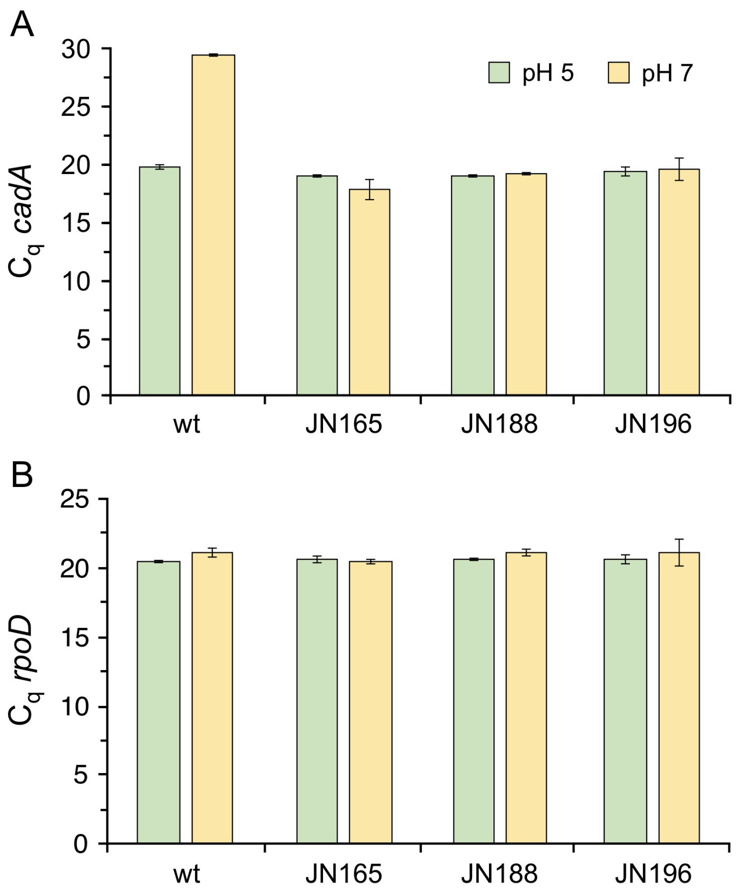

Fig. S16. Expression of *E. coli cadA* and *rpoD* genes at pH 5 and pH 7. Levels of gene expression were measured by qPCR and the quantification cycle (C_q) determined by LinReg for *cadA* and the reference gene *rpoD*. A lower cycle number indicates higher gene expression.

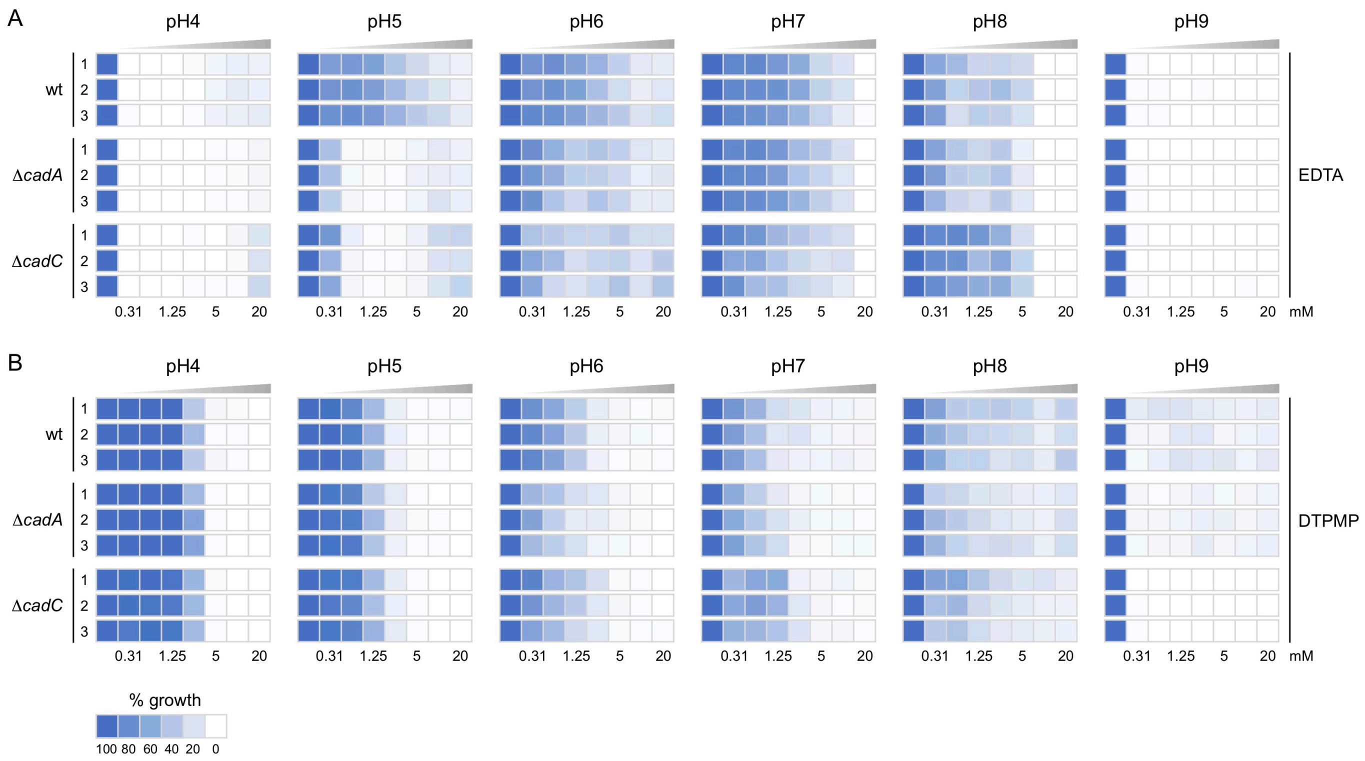

Fig. S17. Susceptibility of *E. coli* wt, *cadA* and *cadC* mutants to EDTA (A) and DTPMP (B) under acidic and alkaline conditions. *E. coli* wt, Δ*cadA* and Δ*cadC* mutants were mixed with serial dilutions of each chelant in LB at the indicated pH and incubated at 37°C with shaking at 150 rpm for 16 h. The extent of growth was measured at $OD_{600\ nm}$ at the endpoint and normalised against controls without chelant to give the percentage growth. Three independent replicates are shown (labelled 1-3), incorporating one of the data sets shown in Figure 8A and B, and are the mean of experiments performed in triplicate with growth indicated by conditional formatting (values capped at 100% growth).

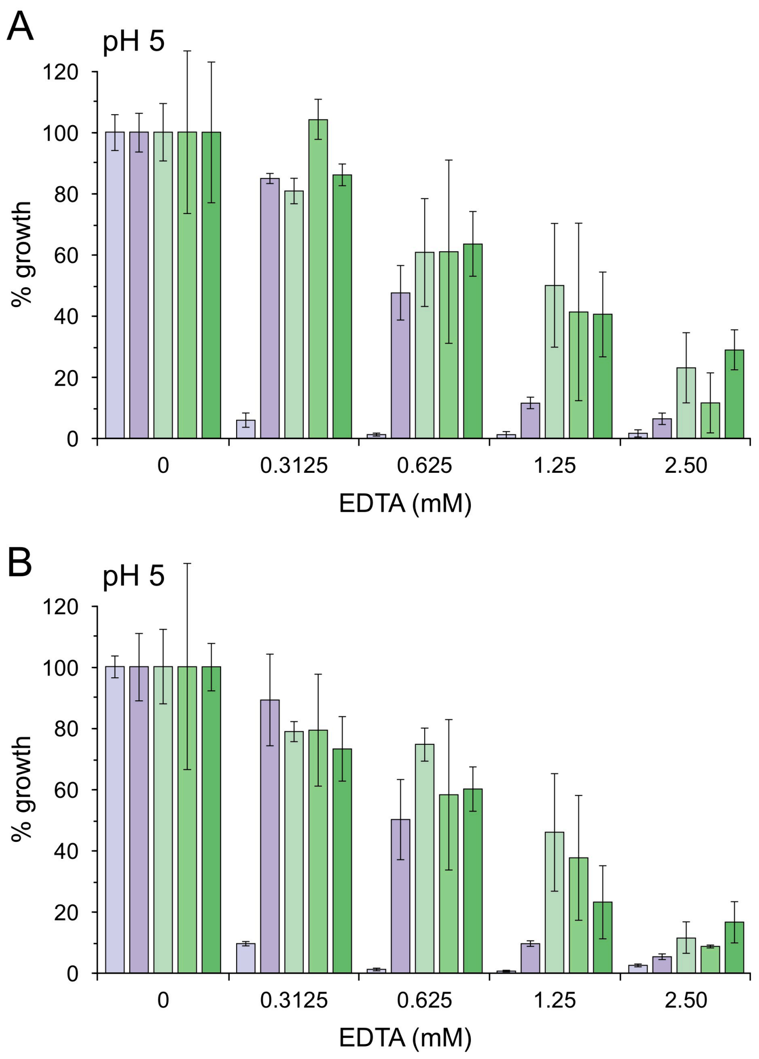

Fig. S18. Effect of plasmid-encoded CadC mutants on *cadC* mutant susceptibility to EDTA at pH 5. The Δ*cadC* mutant transformed with complementing plasmids expressing the CadC wt, mutant variants or the vector control (pET22b) were tested for chelant susceptibility in LB at pH5. Results are independent replicates (A and B) of the data shown in Figure 8C and are the mean and standard deviation of experiments performed in triplicate.

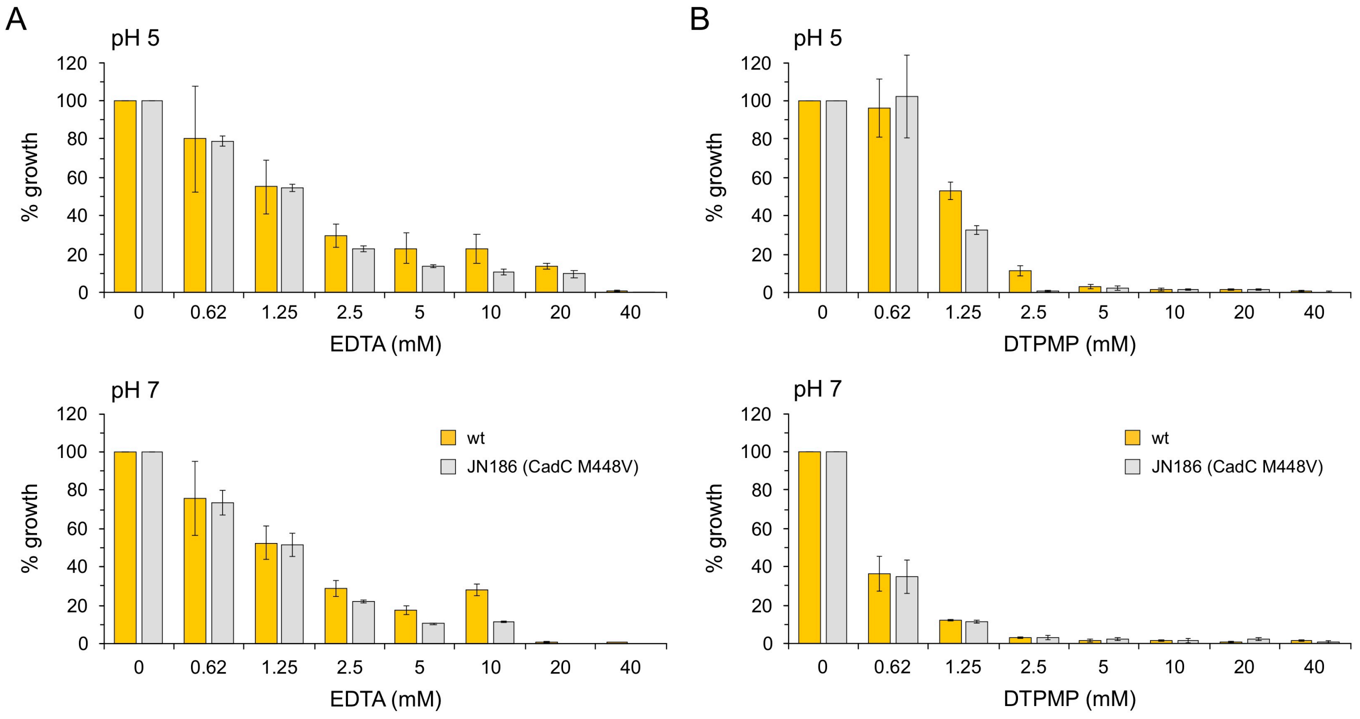

Fig. S19. Susceptibility of *E. coli* JN186 carrying a CadC M448V mutation to EDTA (A) and DTPMP (B) at pH 5 and pH 7. Two-fold serial dilutions of each compound were mixed with each strain and incubated at 37°C with shaking at 150 rpm for 16 h. Growth was measured at $OD_{600\ nm}$ at the end point and normalised against controls without treatment to give the percentage growth. Results represent the mean and standard deviation of three independent experiments.

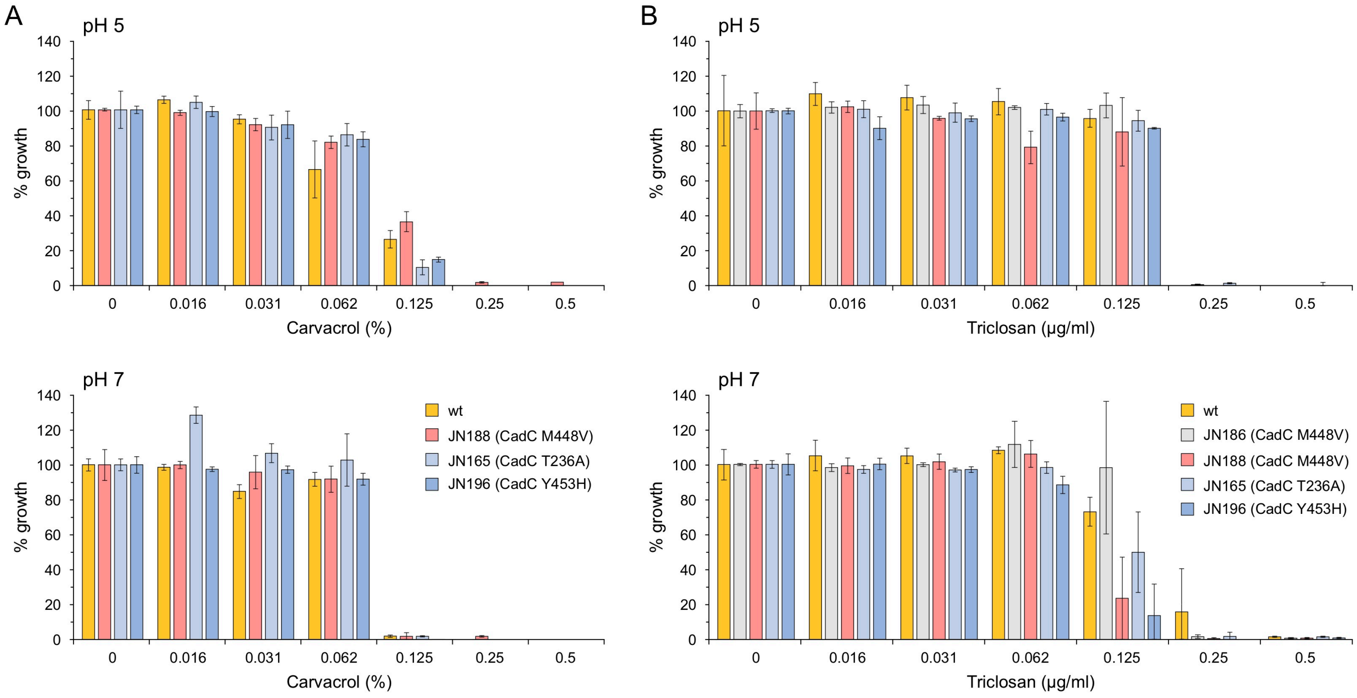

Fig. S20. Susceptibility of *E. coli* chelant-selected strains carrying mutations in *cadC* to carvacrol and triclosan. The sensitivity of strains to carvacrol (A) or triclosan (B) in LB at pH5 and 7 was examined. Two-fold serial dilutions of each compound were mixed with each strain and incubated at 37°C with shaking at 150 rpm for 16 h. Growth was measured at $OD_{600\ nm}$ at the end point and normalised against controls without treatment to give the percentage growth. Results represent the mean and standard deviation of an experiment performed in triplicate. Two additional independent repeats produced similar results.

References

1. Foster AW, Clough SE, Aki Z, Young TR, Clarke AR, Robinson NJ. Metalation calculators for *E. coli* strain JM109 (DE3): aerobic, anaerobic, and hydrogen peroxide exposed cells cultured in LB media. Metallomics. 2022;14:mfac058. doi: https://doi.org/10.1093/mtomcs/mfac058. PubMed PMID: 35933161.
2. Cheng G, Bennett EM, Begley TP, Ealick SE. Crystal structure of 4-amino-5-hydroxymethyl-2-methylpyrimidine phosphate kinase from *Salmonella typhimurium* at 2.3 Å resolution. Structure. 2002;10:225-35. doi: https://doi.org/10.1016/S0969-2126(02)00708-6. PubMed PMID: 11839308.
3. Nodwell MB, Koch MF, Alte F, Schneider S, Sieber SA. A subfamily of bacterial ribokinases utilizes a hemithioacetal for pyridoxal phosphate salvage. J Am Chem Soc. 2014;136:4992-9. doi: https://doi.org/10.1021/ja411785r. PubMed PMID: 24601602.
4. Bateman A. The SIS domain: a phosphosugar-binding domain. Trends Biochem Sci. 1999;24:94-5. doi: https://doi.org/10.1016/S0968-0004(99)01357-2. PubMed PMID: 10203754.
5. Baba T, Ara T, Hasegawa M, Takai Y, Okumura Y, Baba M, et al. Construction of *Escherichia coli* K-12 in-frame, single-gene knockout mutants: the Keio collection. Mol Syst Biol. 2006;2:2006 0008. doi: https://doi.org/10.1038/msb4100050. PubMed PMID: 16738554; PubMed Central PMCID: PMCPMC1681482.
6. Hasman H, Schembri MA, Klemm P. Antigen 43 and type 1 fimbriae determine colony morphology of *Escherichia coli* K-12. J Bacteriol. 2000;182:1089-95. doi: https://doi.org/10.1128/JB.182.4.1089-1095.2000. PubMed PMID: 10648536.
7. Abraham JM, Freitag CS, Clements JR, Eisenstein BI. An invertible element of DNA controls phase variation of type 1 fimbriae of *Escherichia coli*. . Proc Natl Acad Sci USA. 1985;82:5724-7. doi: https://doi.org/10.1073/pnas.82.17.5724. PubMed PMID: 2863818.
8. Paterson JR, Beecroft MS, Mulla RS, Osman D, Reeder NL, Caserta JA, et al. Insights into the antibacterial mechanism of action of chelating agents by selective deprivation of iron, manganese, and zinc. Appl Environ Microbiol. 2022;88:e0164121. doi: https://doi.org/10.1128/AEM.01641-21. PubMed PMID: 34788072.
